# The modified split generalized equilibrium problem for quasi-nonexpansive mappings and applications

**DOI:** 10.1186/s13660-018-1716-9

**Published:** 2018-05-22

**Authors:** Kanyarat Cheawchan, Atid Kangtunyakarn

**Affiliations:** Department of Mathematics, Faculty of Science, King Mongkut’s Institute of Technology Ladkrabang, Bangkok, Thailand

**Keywords:** 47B40, 47H10, 47J20, The modified split generalized equilibrium problem, Quasi-nonexpansive mapping, Variational Inequality problem, Fixed Point problem

## Abstract

In this paper, we introduce a new problem, the modified split generalized equilibrium problem, which extends the generalized equilibrium problem, the split equilibrium problem and the split variational inequality problem. We introduce a new method of an iterative scheme $\{x_{n}\}$ for finding a common element of the set of solutions of variational inequality problems and the set of common fixed points of a finite family of quasi-nonexpansive mappings and the set of solutions of the modified split generalized equilibrium problem without assuming a demicloseness condition and $T_{\omega }:= (1-\omega)I+ \omega T$, where *T* is a quasi-nonexpansive mapping and $\omega \in ( 0,\frac{1}{2} ) $; a difficult proof in the framework of Hilbert space. In addition, we give a numerical example to support our main result.

## Introduction

Let *C* be a nonempty closed convex subset of a real Hilbert space *H*. The set of fixed points of *T* is denoted by $F(T)$. The mapping $T: C \rightarrow C$ is said to be *quasi-nonexpansive* if

$$\Vert Tx-p\Vert \leq \Vert x-p\Vert , $$

for all $x\in C$ and $p\in F(T)$.

### Definition 1.1

([1])

Let $T:H \rightarrow H$. Then the following are equivalent:

1. *T* is firmly nonexpansive,
2. $\Vert Tx-Ty \Vert ^{2} \leq \langle x-y, Tx-Ty \rangle$, $\forall x,y \in H$,
3. $\langle Tx-Ty, (I-T)x-(I-T)y \rangle \geq 0$, $\forall x,y \in H$.

Let $A:C \rightarrow H$ be a mapping. The *variational inequality* is to find a point $u \in C$ such that

1.1 $$ \langle Au, v-u \rangle \geq 0, $$

for all $v \in C$. The set of solutions of (1.1) is denoted by $VI(C,A)$. A mapping $A: C \rightarrow H$ is called *α-inverse strongly monotone* if there exists a positive real number $\alpha > 0$ such that

$$\langle Ax-Ay, x-y \rangle \geq \alpha \Vert Ax-Ay\Vert ^{2}, $$

for all $x,y \in C$. They have been investigated in the literature; see, for example, [2, 3]. Let *F* be a bifunction of $C\times C$ into $\mathbb{R}$, where $\mathbb{R}$ is the set of real numbers. The *equilibrium problem* for $F:C\times C \rightarrow \mathbb{R}$ is to find $x \in C$ such that

1.2 $$ F(x,y)\geq 0,\quad \forall y \in C. $$

The set of solutions of (1.2) is denoted by $EP ( F ) $. Equilibrium problems were introduced by [4] in 1994 and included many well-known problems such as variational inequality, optimization problem, nonexpansive mapping and fixed point problem; see, for example, [5–8].

Let *F* be a function of $C\times C$ into $\mathbb{R}$ and let $f:H \rightarrow H$ be a mapping. The *generalized equilibrium problem* is to find $x\in C$ such that

1.3 $$ F(x,y)+ \bigl\langle f(x),y-x \bigr\rangle \geq 0, $$

for all $y\in C$. The set of solutions of (1.3) is denoted by $EP(F,f)$. When $f\equiv 0$, $EP(F,f)$ is denoted by $EP(F)$ and $F\equiv 0$, $EP(F,f)$ is denoted by $VI(C,f)$.

Throughout this section, let $H_{1}$, $H_{2}$ be real Hilbert spaces and let *C*, *Q* be nonempty closed convex subsets of real Hilbert spaces $H_{1}$ and $H_{2}$, respectively. Let $A:H_{1} \rightarrow H_{2}$ be a bounded linear operator.

In 1994, Censor and Elfving [9] introduced the *split feasibility problem* (in short, SFP) which is to find a point $x \in C$ such that $Ax \in Q$. The set of all solutions of split feasibility problem is denoted by $\varphi = \{x \in C: Ax \in Q\}$.

To solve the SFP, Byrne [10] introduced *CQ* algorithm whose sequence $\{x_{n}\}$ is generated by

$$x_{n+1}=P_{C_{1}} \bigl( x_{n}-\gamma A^{*} ( I-P_{C_{2}} ) Ax _{n} \bigr), $$

where the initial $x_{0} \in H_{1}$ and $\gamma \in (0, 2/L)$, *L* is the spectral radius of the operator $A^{*}A$ and $A^{*}$ is the adjoint of *A*. Then the *CQ* algorithm converges to a solution of the SFP, whenever solutions exist. If there are no solutions of the SFP, the *CQ* algorithm converges to a minimizer of the function

$$\frac{1}{2} \bigl\Vert (I-P_{C_{2}})Ax \bigr\Vert ^{2}, $$

whenever such minimizers exist.

Let $U:H_{1} \rightarrow H_{1}$ and $T: H_{2} \rightarrow H_{2}$ be two nonlinear operators. The *split common fixed points problem* (SCFPP) [11, 12] is to find a point $x^{*}$ such that

$$x^{*} \in F(U)\quad \text{and} \quad Ax^{*} \in F(T). $$

The solution set of SCFPP is denoted by $\Phi =\{p^{*}\in F(U): Ap^{*}\in F(T)\}$. The split common fixed point problem is a generalization of the split feasibility problem.

In 2017, Wang [13] introduced a new method for solving SCFPP as follows:

$$x_{n+1}=x_{n}-\rho_{n} \bigl( ( I-U ) x_{n}+A^{*}(I-T)Ax_{n} \bigr), $$

where $\rho _{n} \subset (0,\infty)$ is chosen such that

1.4 $$ \rho_{n}=\frac{\Vert (I-U)x_{n} \Vert ^{2}+\Vert (I-T)Ax_{n} \Vert ^{2}}{\Vert (I-U)x_{n}+A^{*}(I-T)Ax_{n} \Vert ^{2}} $$

and *U* and *T* are firmly quasi-nonexpansive mappings. Then the sequence $\{x_{n}\}$ converges weakly to *z*, where $z=\lim_{n \rightarrow \infty }P_{\Phi }x_{n}$.

Censor et al. [11, 14] introduced the prototypical *split inverse problem* (SIP) which is a generalization of the split common fixed points problem. In this, there are given two vector spaces *X* and *Y* and a linear operator $A:X \rightarrow Y$. In addition, two inverse problems are involved. The first one, denoted IP_1_, is formulated in the space *X* and the second one, denoted IP_2_, is formulated in the space *Y*. Given these data, the split inverse problem is formulated as follows:

1.5 $$ \text{find a point } x^{*} \in X \text{ that solves IP}_{1}, $$

and such that

1.6 $$ \text{find a point } y^{*} \in Y \text{ that solves IP}_{2}. $$

This problem is used in many modeling arising in sensor networks, radiation therapy treatment planning, color imaging, etc.

The *split equilibrium problem* (SEP) [12] is to find $\widehat{x}\in C$ such that

1.7 $$ F_{1}(\widehat{x},x)\geq 0,\quad \forall x \in C, $$

and such that

1.8 $$ \widehat{y}=A\widehat{x}\in Q \quad \text{solves } F_{2}( \widehat{y},y) \geq 0,\quad \forall y \in Q, $$

where $F_{1}:C\times C\rightarrow \mathbb{R}$ and $F_{2}:Q\times Q \rightarrow \mathbb{R}$ be nonlinear bifunctions. If we consider only problem (1.7), it is the equilibrium problem and we denoted its solution set by $EP(F_{1})$. The solution set of SEP is denoted by $\Gamma = \{\widehat{p} \in EP(F_{1}): A\widehat{p} \in EP(F_{2})\}$. SEP is reduced to $EP(F)$, where $H_{1}\equiv H_{2}$, $F_{1} \equiv F _{2}$ and $A\equiv I$. $EP(F)$ is an unifying model for several problems arising in physics, engineering, science, optimization, economics, etc.

The *split variational inequality problems* (in short, SVIP) were introduced and studied by Cencor et al. [11]: find $\overline{x} \in C$ such that

1.9 $$ \bigl\langle f_{1}(\overline{x}),x-\overline{x} \bigr\rangle \geq 0,\quad \forall x \in C, $$

and such that

1.10 $$ \overline{y}=A\overline{x}\in Q\quad \text{solves } \bigl\langle f_{2}( \overline{y}),y-\overline{y} \bigr\rangle \geq 0,\quad \forall y \in Q, $$

where $f_{1}:C\rightarrow H_{1}$ and $f_{2}:Q\rightarrow H_{2}$ are nonlinear mappings. The solution set of SVIP is denoted by $\Psi = \{ \overline{p} \in VI(C,f_{1}): A\overline{p} \in VI(Q,f_{2})\}$. The split variational inequality problems have already been studied and used in practice as a model in intensity-modulated radiation therapy (IMRT) treatment planning; see, for example, [15] and the modeling of many inverse problems arising for phase retrieval and other real-world problems; for instance, in sensor networks in computerized tomography and data compression; see, for example, [16, 17].

By investigating SEP and SVIP, we introduce the *modified split generalized equilibrium problem* (MSGEP) which is to find $x^{*} \in C$ such that

1.11 $$ F_{1} \bigl(x^{*},x \bigr)+ \bigl\langle f_{1} \bigl(x^{*} \bigr),x-x^{*} \bigr\rangle \geq 0,\quad \forall x \in C, $$

and such that

1.12 $$ y^{*}=Ax^{*}\in Q\quad \text{solves } F_{2} \bigl(y^{*},y \bigr)+ \bigl\langle f_{2} \bigl(y^{*} \bigr),y-y ^{*} \bigr\rangle \geq 0,\quad \forall y \in Q, $$

where $F_{1}:C\times C\rightarrow \mathbb{R}$ and $F_{2}:Q\times Q \rightarrow \mathbb{R}$ are nonlinear bifunctions and $f_{1}: C \rightarrow H_{1}$ and $f_{2}: Q \rightarrow H_{2}$ are nonlinear mappings. The solution set of MSGEP is denoted by $\Omega =\{p^{*} \in EP(F_{1},f_{1}): Ap^{*}\in EP(F_{2},f_{2})\}$.

### Remark 1.1

1. If we put $f_{1} \equiv f_{2} \equiv 0$ in MSGEP then the MSGEP is reduced to SEP.
2. If we put $F_{1} \equiv F_{2} \equiv 0$ in MSGEP then the MSGEP is reduced to SVIP.
3. In the case of bifunctions $F_{1}$ and $F_{2}$ are according to (A1)–(A4). From (1.11), (1.12) and Lemma 2.2, we have $x^{*} \in F(T^{F_{1}}_{r} ( I-rf_{1} ))$ and $Ax^{*} \in F(T^{F_{2}}_{s} ( I-sf_{2} ))$, for all $r, s>0$. So, MSGEP can be viewed as SCFPP.

MSGEP is a generalization of the generalized equilibrium problem, the split equilibrium problem and the split variational inequality problem. So, this problem can be used in sensor networks, data compression, practice as a model in intensity-modulated radiation therapy (IMRT) treatment planning, robustness to marginal changes and equilibrium stability etc.

### Example 1.2

Let $H_{1}=[0, 6]$, $H_{2}=[0, 18]$, $C=[2, 5]$ and $Q=[6, 10]$. Let $A : H_{1} \rightarrow H_{2}$ be defined by $Ax = 3x$ for all $x \in H_{1}$. Let the mapping $F_{1}: C \times C \rightarrow \mathbb{R}$ be defined by

$$F_{1} \bigl(x^{*}, x \bigr) = - \bigl(x^{*}-2 \bigr)^{2}+(x-2)^{2},\quad \forall x, y \in C, $$

and $F_{2}: Q \times Q \rightarrow \mathbb{R}$ be defined by

$$F_{2} \bigl(y^{*}, y \bigr) = - \bigl(y^{*}-6 \bigr)^{2}+(y-6)^{2},\quad \forall x, y \in Q. $$

Let the mapping $f_{1}: C \rightarrow H_{1}$ be defined by $f_{1}x = \frac{x-2}{9}$, $\forall x\in C$ and the mapping $f_{2}: Q \rightarrow H_{2}$ be defined by $f_{2}x = \frac{x-6}{7}$, $\forall x\in Q$.

Then $2 \in \Omega $. Therefore 2 is a solution of MSGEP.

In 2012, Tain and Jin [18] introduced iterative algorithms involving a quasi-nonexpansive mapping. They generated the iterative as follows:

$$x_{n+1}=\alpha_{n}\gamma f(x_{n})+(I- \alpha_{n}A)T_{\omega }x_{n}, $$

where *A* is a bounded linear operator on *H*, *T* is a quasi-nonexpansive mapping on *H*, *f* is a contraction with coefficient *a* under suitable conditions of the parameters $\alpha_{n}$, *γ* and *ω*. By assuming $\omega \in ( 0,\frac{1}{2} ) $, $T_{\omega }:= (1-\omega)I+\omega T$ and *T* is demiclosed on *H*.

Motivated by SFP and SVIP, we introduced a new problem, the modified split generalized equilibrium problem, which extends the generalized equilibrium problem, the split equilibrium problem and the split variational inequality problem. Many authors proved strong convergence theorem involving a quasi-nonexpansive mapping *T* by assuming $T_{\omega }:= (1-\omega)I+\omega T$ and *T* is demiclosed on *H*; a difficult proof. Motivated by [19], we introduced Remark 2.5 and [11, 12] and [18], we introduce a new method of iterative scheme $\{x_{n}\}$ for finding a common element of the set of solutions of variational inequality problems and the set of common fixed points of a finite family of quasi-nonexpansive mappings and the set of solutions of the modified split generalized equilibrium problem without the condition above in the framework of a Hilbert space.

## Preliminaries

Let *H* be a real Hilbert space with inner product $\langle \cdot, \cdot \rangle $ and norm $\Vert \cdot \Vert $. Throughout this paper, we use the notations of weak and strong convergence by “⇀” and “→”*Opial’s condition* [20], i.e., for any sequence $\{ x_{n} \} $ with $x_{n} \rightharpoonup x$, the inequality $\lim_{n \rightarrow \infty } \inf \Vert x_{n}-x \Vert < \lim_{n \rightarrow \infty } \inf \Vert x_{n}-y \Vert $, holds for every $y \in H$ with $y \neq x$.

For solving the equilibrium problem, we assume that the bifunction $F:C\times C\rightarrow \mathbb{R}$ satisfy the following conditions:

1. $F(x,x)=0$ for all $x\in C$,
2. *F* is monotone, i.e., $F(x,y)+F(y,x)\leq 0$ for all $x,y\in C$,
3. for each $x,y,z\in C$, $\lim_{t \downarrow 0} F ( tz+(1-t)x,y ) \leq F(x,y)$,
4. for each $x\in C$, $y\mapsto F(x,y)$ is convex and lower semicontinuous.

### Lemma 2.1

([4])

*Let* *C* *be a nonempty closed convex subset of* *H* *and let* *F* *be a bifunction of* $C \times C$ *into* $\mathbb{R}$ *satisfying* (A1)*–*(A4). *Let* $r>0$ *and* $x \in H$. *Then there exists* $z \in C$ *such that*

$$F(z,y) + \frac{1}{r} \langle y-z, z-x \rangle \geq 0,\quad \forall y \in C. $$

### Lemma 2.2

([21])

*Assume that* $F:C\times C\rightarrow \mathbb{R}$ *satisfies* (A1)*–*(A4). *For* $r >0$, *define a mapping* $T_{r}:H\rightarrow C$ *as follows*:

$$\begin{aligned} T_{r}(x)= \biggl\{ z\in C : F(z,y)+\frac{1}{r}\langle y-z,z-x \rangle \geq 0,\forall y\in C \biggr\} \end{aligned}$$

*for all* $x\in H$. *Then the following hold*:

1. $T_{r}$ *is single*-*valued*,
2. $T_{r}$ *is firmly nonexpansive*, *i*.*e*., *for any* $x,y \in H$,
  $$\bigl\Vert T_{r}(x)-T_{r}(y) \bigr\Vert ^{2} \leq \bigl\langle T_{r}(x)-T _{r}(y),x-y \bigr\rangle , $$
3. $F ( T_{r} ) = EP(F)$,
4. $EP(F)$ *is closed and convex*.

### Lemma 2.3

([22])

*Let* *H* *be a real Hilbert space*, *let* *C* *be a nonempty closed convex subset of* *H* *and let* *A* *be a mapping of* *C* *into* *H*. *Let* $u \in C$. *Then*, *for* $\lambda > 0$,

$$u = P_{C} ( I-\lambda A ) u \quad \Leftrightarrow \quad u \in VI ( C,A ), $$

*where* $P_{C}$ *is the metric projection of* *H* *onto* *C*.

### Lemma 2.4

*Let* *C* *be a nonempty closed convex subset of a real Hilbert space* *H*. *Let* $\{ T_{i} \} ^{N}_{i=1}$ *be a finite family of quasi*-*nonexpansive mappings of* *C* *into* *H* *with* $\bigcap^{N}_{i=1} F ( T_{i} ) \neq \emptyset $ *and let* $0 < a_{i} < 1 $ *with* $\sum^{N}_{i=1}a_{i}=1$. *Then*

$$\bigcap^{N}_{i=1} F ( T_{i} ) = VI \Biggl( C,\sum^{N}_{i=1}a_{i} ( I-T_{i} ) \Biggr). $$

### Proof

In this lemma, we show that $\bigcap^{N}_{i=1} F ( T_{i} ) = \bigcap^{N}_{i=1} VI ( C,I-T _{i} ) $ and $\bigcap^{N}_{i=1} VI ( C,I-T_{i} ) = VI ( C,\sum^{N}_{i=1}a _{i} ( I-T_{i} ) )$. Lastly, we have

$$\bigcap^{N}_{i=1} F ( T_{i} ) = VI \Biggl( C,\sum^{N}_{i=1}a_{i} ( I-T_{i} ) \Biggr). $$

To start with, it is easy to see that $\bigcap^{N}_{i=1} F ( T_{i} ) \subseteq \bigcap^{N}_{i=1} VI ( C,I-T_{i} )$. Next, we show that $\bigcap^{N}_{i=1} VI ( C,I-T_{i} ) \subseteq \bigcap^{N}_{i=1} F ( T_{i} )$. Let $u \in \bigcap^{N}_{i=1} VI ( C,I-T_{i} ) $ and $\bigcap^{N}_{i=1} F ( T_{i} ) \neq \emptyset$. So, we get $u \in VI ( C,I-T_{i} )$, $\forall i=1, 2, \ldots,N$. We may write

2.1 $$ \bigl\langle u-v,(I-T_{i})u \bigr\rangle \leq 0,\quad \forall v \in C. $$

There exists $v^{*}\in C $ such that $v^{*}=T_{i}v^{*}$, $\forall i=1, 2, \ldots,N$. Since $T_{i}$ is a quasi-nonexpansive mapping, $\forall i=1, 2, \ldots,N$, it follows that

2.2 $$\begin{aligned} \bigl\Vert T_{i}u-v^{*} \bigr\Vert ^{2} &= \bigl\Vert \bigl( u-v^{*} \bigr) - ( I-T _{i} ) u \bigr\Vert ^{2} \\ &= \bigl\Vert u-v^{*} \bigr\Vert ^{2} - 2 \bigl\langle u-v^{*}, ( I-T_{i} ) u \bigr\rangle + \bigl\Vert ( I-T_{i} ) u \bigr\Vert ^{2} \\ &\leq \bigl\Vert u-v^{*} \bigr\Vert ^{2}. \end{aligned}$$

By using (2.1) and (2.2), we conclude that

$$\bigl\Vert ( I-T_{i} ) u \bigr\Vert ^{2} \leq 2 \bigl\langle u-v^{*}, ( I-T_{i} ) u \bigr\rangle \leq 0. $$

It implies that $u \in \bigcap^{N}_{i=1} F ( T_{i} )$. Therefore $\bigcap^{N}_{i=1} VI ( C,I-T_{i} ) \subseteq \bigcap^{N}_{i=1} F ( T_{i} ) $. Hence

$$\bigcap^{N}_{i=1} F ( T_{i} ) = \bigcap^{N}_{i=1} VI ( C,I-T _{i} ). $$

After that, we show $\bigcap^{N}_{i=1} VI ( C,I-T_{i} ) = VI ( C,\sum^{N}_{i=1}a _{i} ( I-T_{i} ) ) $ where $0 < a_{i} < 1 $ and $\sum^{N}_{i=1}a_{i}=1$. Observe that

$$\begin{aligned}& u \in \bigcap^{N}_{i=1} VI ( C,I-T_{i} ) \\& \quad \Leftrightarrow \quad u \in VI ( C,I-T_{i} ),\quad \forall i=1, 2, \ldots,N \\& \quad \Leftrightarrow \quad \bigl\langle ( I-T_{i} ) u,v-u \bigr\rangle \geq 0,\quad \forall v\in C \text{ and } \forall i=1, 2, \ldots,N \\& \quad \Leftrightarrow \quad \sum^{N}_{i=1} a_{i} \bigl\langle ( I-T_{i} ) u,v-u \bigr\rangle \geq 0, \quad \forall v\in C \\& \quad \Leftrightarrow \quad \Biggl\langle \sum^{N}_{i=1} a_{i} ( I-T_{i} ) u,v-u \Biggr\rangle \geq 0,\quad \forall v\in C \\& \quad \Leftrightarrow \quad u \in VI \Biggl( C,\sum ^{N}_{i=1}a_{i} ( I-T_{i} ) \Biggr). \end{aligned}$$

Therefore $\bigcap^{N}_{i=1} VI ( C,I-T_{i} ) = VI ( C,\sum^{N}_{i=1}a _{i} ( I-T_{i} ) )$. Hence $\bigcap^{N}_{i=1} F ( T _{i} ) = VI ( C,\sum^{N}_{i=1}a_{i} ( I-T_{i} ) )$. □

### Remark 2.5

From Lemma 2.3 and Lemma 2.4, we have

$$\bigcap^{N}_{i=1} F ( T_{i} ) = VI \Biggl( C,\sum^{N}_{i=1}a_{i} ( I-T_{i} ) \Biggr) = F \Biggl( P_{C} \Biggl( I-\lambda \Biggl( \sum^{N}_{i=1}a_{i} ( I-T_{i} ) \Biggr) \Biggr) \Biggr), $$

for all $\lambda > 0$ and $0 < a_{i} < 1$ with $\sum^{N}_{i=1}a_{i}=1$.

### Lemma 2.6

([23])

*Let* $\{ s_{n} \} $ *be a sequence of nonnegative real numbers satisfying*

$$s_{n+1} \leq ( 1- \alpha_{n} ) s_{n} + \delta_{n},\quad \forall n \geq 0, $$

*where* $\{ \alpha_{n} \} $ *is a sequence in* $( 0,1 ) $ *and* $\{ \delta_{n} \} $ *is a sequence such that*

$$(1)\quad \sum_{n=1}^{\infty } \alpha_{n} = \infty,\qquad (2)\quad \limsup_{n \rightarrow \infty } \frac{\delta_{n}}{\alpha_{n}} \leq 0\quad \textit{or}\quad \sum_{n=1}^{\infty } \vert \delta_{n} \vert < \infty. $$

*Then* $\lim_{n \rightarrow \infty } s_{n} = 0$.

## Main results

### Lemma 3.1

*Let* *C* *and* *Q* *be nonempty closed convex subsets of a real Hilbert spaces* $H_{1}$ *and* $H_{2}$, *respectively*. *Let* $A:H_{1} \rightarrow H _{2}$ *be a bounded linear operator*. *Let* $F_{1}:C \times C \rightarrow \mathbb{R}$ *and* $F_{2}:Q \times Q \rightarrow \mathbb{R}$ *be the bifunctions satisfying* (A1)*–*(A4). *Let* $f_{1}: H_{1} \rightarrow H _{1}$ *be a* *ρ*-*inverse strongly monotone mapping and* $f_{2}: H _{2} \rightarrow H_{2}$ *be a firmly nonexpansive mapping*. *Then*

1. $T^{F_{1}}_{r}(I-rf_{1})$ *and* $T^{F_{2}}_{s} ( I-sf_{2} ) $ *are nonexpansive mapping*,
2. $$\begin{aligned} &\bigl\Vert T^{F_{1}}_{r} ( I-rf_{1} ) \bigl( p+ \gamma A^{*} \bigl( T^{F_{2}}_{s} ( I-sf_{2} ) -I \bigr) Ap \bigr) \\ &\qquad {}- T^{F_{1}}_{r} ( I-rf_{1} ) \bigl( q+ \gamma A^{*} \bigl( T^{F_{2}}_{s} ( I-sf_{2} ) -I \bigr) Aq \bigr) \bigr\Vert ^{2} \\ &\quad \leq \Vert p-q \Vert ^{2}+\gamma (\gamma L-1) \bigl\Vert \bigl( T ^{F_{2}}_{s} ( I-sf_{2} ) -I \bigr) Ap- \bigl( T^{F_{2}}_{s} ( I-sf_{2} ) -I \bigr) Aq \bigr\Vert ^{2}, \end{aligned}$$

*for all* $p, q \in C$, *where* $r \in (0,2\rho)$, $s \in (0,1)$, $\gamma \in (0,1/L)$, *L* *is the spectral radius of the operator* $A^{*}A$ *and* $A^{*}$ *is the adjoint of* *A*, $T^{F_{1}}_{r}:H_{1}\rightarrow C$ *defined by*

$$\begin{aligned} T^{F_{1}}_{r}(x)= \biggl\{ z\in C : F_{1}(z,y)+ \frac{1}{r}\langle y-z,z-x \rangle \geq 0,\forall y\in C \biggr\} , \end{aligned}$$

*for all* $x\in H_{1}$ *and* $T^{F_{2}}_{s}:H_{2}\rightarrow Q$ *defined by*

$$\begin{aligned} T^{F_{2}}_{s}(\overline{x})= \biggl\{ \overline{z}\in Q : F_{2}( \overline{z},y)+\frac{1}{s}\langle y-\overline{z}, \overline{z}- \overline{x}\rangle \geq 0,\forall y\in Q \biggr\} , \end{aligned}$$

*for all* $\overline{x}\in H_{2}$.

### Proof

Let $p, q \in C$. First, we show 1 is true. Since $f_{1}$ is a *ρ*-inverse strongly monotone mapping and $r \in (0,2\rho)$, we obtain

$$\begin{aligned} \bigl\Vert T^{F_{1}}_{r}(I-rf_{1})p-T^{F_{1}}_{r}(I-rf_{1})q \bigr\Vert ^{2} & \leq \Vert p-q\Vert ^{2}-2r \langle p-q,f_{1}p-f_{1}q \rangle +r ^{2}\Vert f_{1}p-f_{1}q\Vert ^{2} \\ &\leq \Vert p-q \Vert ^{2} + r(r-2\rho)\Vert f_{1}p-f_{1}q \Vert ^{2} \\ &\leq \Vert p-q \Vert ^{2}. \end{aligned}$$

Thus $T^{F_{1}}_{r}(I-rf_{1})$ is a nonexpansive mapping. Since $f_{2}$ is a firmly nonexpansive mapping and $s \in (0,1)$, we get

$$\begin{aligned} \bigl\Vert T^{F_{2}}_{s} ( I-sf_{2} ) \overline{p}-T^{F_{2}}_{s} ( I-sf_{2} ) \overline{q} \bigr\Vert ^{2} &\leq \Vert \overline{p}-\overline{q} \Vert ^{2} - 2s\langle \overline{p}- \overline{q}, f_{2} \overline{p}-f_{2}\overline{q}\rangle +s^{2} \Vert f_{2}\overline{p}-f_{2}\overline{q} \Vert ^{2} \\ &\leq \Vert \overline{p}-\overline{q} \Vert ^{2} - s(2-s)\Vert f_{2}\overline{p}-f_{2}\overline{q} \Vert ^{2} \\ &\leq \Vert \overline{p}-\overline{q} \Vert ^{2}, \end{aligned}$$

for all $\overline{p}, \overline{q}\in Q$. Therefore $T^{F_{2}}_{s} ( I-sf_{2} ) $ is a nonexpansive mapping.

Next, we show 2 is true. From Lemma 3.1(1), we have

3.1 $$\begin{aligned} & \bigl\Vert T^{F_{1}}_{r} ( I-rf_{1} ) \bigl( p+\gamma A^{*} \bigl( T^{F_{2}}_{s} ( I-sf_{2} ) -I \bigr) Ap \bigr) \\ &\qquad {}- T^{F_{1}}_{r} ( I-rf_{1} ) \bigl( q+ \gamma A^{*} \bigl( T^{F_{2}}_{s} ( I-sf_{2} ) -I \bigr) Aq \bigr) \bigr\Vert ^{2} \\ & \quad \leq \bigl\Vert ( p - q ) +\gamma \bigl( A^{*} \bigl( T^{F _{2}}_{s} ( I-sf_{2} ) -I \bigr) Ap - A^{*} \bigl( T^{F_{2}}_{s} ( I-sf_{2} ) -I \bigr) Aq \bigr) \bigr\Vert ^{2} \\ &\quad \leq \Vert p-q \Vert ^{2}+2\gamma \bigl\langle Ap-Aq, \bigl( T ^{F_{2}}_{s} ( I-sf_{2} ) -I \bigr) Ap- \bigl( T^{F_{2}}_{s} ( I-sf_{2} ) -I \bigr) Aq \bigr\rangle \\ &\qquad {}+\gamma^{2}L \bigl\Vert \bigl( T^{F_{2}}_{s} ( I-sf_{2} ) -I \bigr) Ap- \bigl( T^{F_{2}}_{s} ( I-sf_{2} ) -I \bigr) Aq \bigr\Vert ^{2}. \end{aligned}$$

From the property of $T^{F_{2}}_{s}$, we get

3.2 $$\begin{aligned} & \bigl\Vert ( I-sf_{2} ) Ap- ( I-sf_{2} ) Aq \bigr\Vert ^{2} \\ &\quad \geq \bigl\Vert T^{F_{2}}_{s} ( I-sf_{2} ) Ap - T^{F_{2}} _{s} ( I-sf_{2} ) Aq-(Ap-Aq)+(Ap-Aq) \bigr\Vert ^{2} \\ &\quad = \bigl\Vert \bigl( T^{F_{2}}_{s} ( I-sf_{2} ) -I \bigr) Ap - \bigl( T^{F_{2}}_{s} ( I-sf_{2} ) -I \bigr) Aq \bigr\Vert ^{2} \\ &\qquad {}+ 2 \bigl\langle \bigl( T^{F_{2}}_{s} ( I-sf_{2} ) -I \bigr) Ap - \bigl( T^{F_{2}}_{s} ( I-sf_{2} ) -I \bigr) Aq, Ap-Aq \bigr\rangle \\ &\qquad {}+ \Vert Ap-Aq \Vert ^{2}. \end{aligned}$$

We have

3.3 $$\begin{aligned} & \bigl\Vert ( I-sf_{2} ) Ap- ( I-sf_{2} ) Aq \bigr\Vert ^{2} \\ &\quad = \Vert Ap-Aq \Vert ^{2}-2s\langle Ap-Aq, f_{2}Ap-f_{2}Aq \rangle \\ &\qquad {}+ s^{2}\Vert f_{2}Ap-f_{2}Aq \Vert ^{2}. \end{aligned}$$

From (3.2), (3.3) and the property of firmly nonexpansive mapping, we get

$$\begin{aligned} &2 \bigl\langle \bigl( T^{F_{2}}_{s} ( I-sf_{2} ) -I \bigr) Ap - \bigl( T^{F_{2}}_{s} ( I-sf_{2} ) -I \bigr) Aq, Ap-Aq \bigr\rangle \\ &\quad \leq - \bigl\Vert \bigl( T^{F_{2}}_{s} ( I-sf_{2} ) -I \bigr) Ap - \bigl( T^{F_{2}}_{s} ( I-sf_{2} ) -I \bigr) Aq \bigr\Vert ^{2} \\ &\qquad {}-2s\langle Ap-Aq, f_{2}Ap-f_{2}Aq\rangle + s^{2} \Vert f_{2}Ap-f _{2}Aq \Vert ^{2} \\ &\quad \leq - \bigl\Vert \bigl( T^{F_{2}}_{s} ( I-sf_{2} ) -I \bigr) Ap - \bigl( T^{F_{2}}_{s} ( I-sf_{2} ) -I \bigr) Aq \bigr\Vert ^{2}. \end{aligned}$$

That is,

3.4 $$\begin{aligned} &2\gamma \bigl\langle \bigl( T^{F_{2}}_{s} ( I-sf_{2} ) -I \bigr) Ap - \bigl( T^{F_{2}}_{s} ( I-sf_{2} ) -I \bigr) Aq, Ap-Aq \bigr\rangle \\ &\quad \leq -\gamma \bigl\Vert \bigl( T^{F_{2}}_{s} ( I-sf_{2} ) -I \bigr) Ap - \bigl( T^{F_{2}}_{s} ( I-sf_{2} ) -I \bigr) Aq \bigr\Vert ^{2}. \end{aligned}$$

Substituting (3.4) in (3.1), we obtain

$$\begin{aligned} & \bigl\Vert T^{F_{1}}_{r} ( I-rf_{1} ) \bigl( p+ \gamma A^{*} \bigl( T^{F_{2}}_{s} ( I-sf_{2} ) -I \bigr) Ap \bigr) \\ &\qquad {}- T^{F_{1}}_{r} ( I-rf_{1} ) \bigl( q+ \gamma A^{*} \bigl( T^{F_{2}}_{s} ( I-sf_{2} ) -I \bigr) Aq \bigr) \bigr\Vert ^{2} \\ &\quad \leq \Vert p-q \Vert ^{2} -\gamma \bigl\Vert \bigl( T^{F_{2}}_{s} ( I-sf_{2} ) -I \bigr) Ap - \bigl( T^{F_{2}}_{s} ( I-sf _{2} ) -I \bigr) Aq \bigr\Vert ^{2} \\ &\qquad {}+\gamma^{2}L \bigl\Vert \bigl( T^{F_{2}}_{s} ( I-sf_{2} ) -I \bigr) Ap- \bigl( T^{F_{2}}_{s} ( I-sf_{2} ) -I \bigr) Aq \bigr\Vert ^{2} \\ &\quad = \Vert p-q \Vert ^{2}+\gamma (\gamma L-1) \bigl\Vert \bigl( T^{F _{2}}_{s} ( I-sf_{2} ) -I \bigr) Ap- \bigl( T^{F_{2}}_{s} ( I-sf_{2} ) -I \bigr) Aq \bigr\Vert ^{2}. \end{aligned}$$

 □

### Lemma 3.2

*Let* *C* *be a nonempty closed convex subset of a real Hilbert space* *H* *and let* $T: C\rightarrow C$ *be a quasi*-*nonexpansive mapping with* $F ( T ) \neq \emptyset $. *Then*

$$\bigl\Vert (I-T)x \bigr\Vert ^{2} \leq 2 \bigl\langle x-z, (I-T)x \bigr\rangle ,\quad \forall x \in C. $$

### Proof

Let $x \in C$ and $z \in F ( T ) $. Since *T* is a quasi-nonexpansive mapping, we get

$$\begin{aligned} \Vert Tx-z \Vert ^{2} &= \bigl\Vert (x-z)-(I-T)x \bigr\Vert ^{2} \\ &=\Vert x-z\Vert ^{2} - 2 \bigl\langle x-z, (I-T)x \bigr\rangle + \bigl\Vert (I-T)x \bigr\Vert ^{2} \\ &\leq \Vert x-z \Vert ^{2}. \end{aligned}$$

We can conclude that

$$\bigl\Vert (I-T)x \bigr\Vert ^{2} \leq 2 \bigl\langle x-z, (I-T)x \bigr\rangle . $$

 □

### Lemma 3.3

*Let* *C* *be a nonempty closed convex subset of a real Hilbert space* *H*. *Let* $\{ T_{i} \} ^{N}_{i=1}$ *be a finite family of quasi*-*nonexpansive mappings of* *C* *into itself with* $\bigcap^{N}_{i=1} F ( T_{i} ) \neq \emptyset $. *Then*

$$\Biggl\Vert P_{C} \Biggl( I-\overline{\lambda } \Biggl( \sum ^{N}_{i=1}k_{i} ( I-T_{i} ) \Biggr) \Biggr) x-z \Biggr\Vert ^{2}\leq \Vert x-z \Vert ^{2}, $$

*for all* $x \in C$, *where* $0 < k_{i} < 1$ *with* $\sum^{N}_{i=1}k_{i}=1$ *and* $0 < \overline{\lambda } < 1$.

### Proof

Let $x \in C$ and $z \in \bigcap^{N}_{i=1} F ( T_{i} ) $. From Remark 2.5 and $z\in \bigcap^{N}_{i=1} F ( T_{i} ) $, we have $z \in F ( P_{C} ( I-\overline{\lambda } ( \sum^{N}_{i=1}k_{i} ( I-T_{i} ) ) ) ) $ and $z=T_{i}z$, $\forall i=1, 2, \ldots, N$. Since $P_{C}$ is nonexpansive mapping, $0 < \overline{\lambda } < 1$ and Lemma 3.2, we have

3.5 $$\begin{aligned} & \Biggl\Vert P_{C} \Biggl( I-\overline{\lambda } \Biggl( \sum^{N}_{i=1}k_{i} ( I-T_{i} ) \Biggr) \Biggr) x-z \Biggr\Vert ^{2} \\ &\quad = \Biggl\Vert P_{C} \Biggl( I-\overline{\lambda } \Biggl( \sum^{N}_{i=1}k _{i} ( I-T_{i} ) \Biggr) \Biggr) x-P_{C} \Biggl( I-\overline{ \lambda } \Biggl( \sum^{N}_{i=1}k_{i} ( I-T_{i} ) \Biggr) \Biggr) z \Biggr\Vert ^{2} \\ &\quad \leq \Vert x-z \Vert ^{2}-2\overline{\lambda }\sum ^{N}_{i=1}k _{i} \bigl\langle x-z, ( I-T_{i} ) x \bigr\rangle + \overline{ \lambda }^{2}\sum ^{N}_{i=1}k_{i} \bigl\Vert ( I-T_{i} ) x \bigr\Vert ^{2} \\ &\quad \leq \Vert x-z \Vert ^{2}-\overline{\lambda }\sum ^{N}_{i=1}k _{i} \bigl\Vert ( I-T_{i} ) x \bigr\Vert ^{2} + \overline{\lambda } ^{2}\sum^{N}_{i=1}k_{i} \bigl\Vert ( I-T_{i} ) x \bigr\Vert ^{2} \\ &\quad \leq \Vert x-z \Vert ^{2}. \end{aligned}$$

 □

Next, we prove a strong convergence theorem for solving the modified split generalized equilibrium problem (MSGEP).

### Theorem 3.4

*Let* *C* *and* *Q* *be nonempty closed convex subsets of a real Hilbert spaces* $H_{1}$ *and* $H_{2}$, *respectively*. *Let* $A:H_{1} \rightarrow H _{2}$ *be a bounded linear operator*. *Let* $D_{1}, D_{2}:C \rightarrow H _{1}$ *be* *α*, *β*-*inverse strongly monotone mappings*, *respectively*. *Let* $F_{1}:C \times C \rightarrow \mathbb{R}$ *and* $F_{2}:Q \times Q \rightarrow \mathbb{R}$ *be the bifunctions satisfying* (A1)*–*(A4). *Let* $\{ T_{i} \} ^{N}_{i=1}$ *be a finite family of quasi*-*nonexpansive mappings of* *C* *into itself with* $\bigcap^{N}_{i=1} F ( T_{i} ) \neq \emptyset $. *Let* $f_{1}: H_{1} \rightarrow H_{1}$ *be a* *ρ*-*inverse strongly monotone mapping and* $f_{2}: H_{2} \rightarrow H_{2}$ *be a firmly nonexpansive mapping*. *Assume* $\mathcal{F}= VI(C,D_{1}) \cap VI(C,D_{2}) \cap \bigcap^{N}_{i=1} F ( T_{i} ) \cap \Omega \neq \emptyset $. *For given* $x_{1},u\in C$ *and let* $\{ x_{n} \}$, $\{ u_{n} \}$ *and* $\{ y_{n} \}$ *be sequences generated by*

3.6 $$ \textstyle\begin{cases} u_{n} = T^{F_{1}}_{r} ( I-rf_{1} ) ( x_{n}+\gamma A ^{*} ( T^{F_{2}}_{s} ( I-sf_{2} ) -I ) Ax_{n} ), \\ y_{n} = P_{C} ( I-d_{1}D_{1} ) ( au_{n}+ ( 1-a ) P_{C} ( I-d_{2}D_{2} ) u_{n} ), \\ x_{n+1} = \alpha_{n}u + \beta_{n}x_{n} + \gamma_{n}P_{C} ( I - \lambda_{n} ( \sum^{N}_{i=1}k_{i} ( I-T_{i} ) ) ) y _{n}, \quad \forall n\in \mathbb{N}, \end{cases} $$

*where* $d_{1} \in (0,2\alpha)$, $d_{2} \in (0,2\beta)$, $r \in (0,2 \rho)$, $s \in (0,1)$, $a \in [0,1]$, $0 < k_{i} < 1$ *with* $\sum^{N}_{i=1}k_{i}=1$, $\gamma \in (0,1/L)$, *L* *is the spectral radius of the operator* $A^{*}A$ *and* $A^{*}$ *is the adjoint of* *A*. *Also* $\{ \alpha_{n} \}$, $\{ \beta_{n} \}$, $\{ \gamma_{n} \}$ *are sequences in* $[0,1]$ *with* $\alpha_{n} + \beta_{n} + \gamma_{n} = 1$ *for all* $n \in \mathbb{N}$. *Suppose the following conditions hold*:

(i) $\lim_{n \rightarrow \infty } \alpha_{n} =0$ *and* $\sum_{n=1}^{\infty } \alpha_{n} = \infty $,
(ii) $0 < c \leq \beta_{n}, \gamma_{n} \leq d < 1 $ *for some* $c, d > 0$ *for all* $n \geq 1$,
(iii) $\sum_{n=1}^{\infty } \lambda_{n} < \infty$ *and* $0 < \lambda_{n} < 1 $,
(iv) $\sum_{n=1}^{\infty } \vert \alpha_{n+1} - \alpha_{n} \vert < \infty$, $\sum_{n=1}^{\infty } \vert \beta_{n+1} - \beta_{n} \vert < \infty $.

*Then* $\{ x_{n} \}$, $\{ u_{n} \}$ *and* $\{ y_{n} \}$ *converge strongly to* $z=P_{\mathcal{F}}u$.

### Proof

Let $x, y \in C$ and $z \in \mathcal{F}$. First, we show that $( I-d_{1}D_{1} ) $ is a nonexpansive mapping. Since $D_{1}$ is an *α*-inverse strongly monotone mapping, we obtain

$$\begin{aligned} \bigl\Vert (I-d_{1}D_{1})x-(I-d_{1}D_{1})y \bigr\Vert ^{2}&=\Vert x-y\Vert ^{2}-2d_{1} \langle x-y,D_{1}x-D_{1}y \rangle +d _{1}^{2} \Vert D_{1}x-D_{1}y\Vert ^{2} \\ & \leq \Vert x-y \Vert ^{2} + d_{1}(d_{1}-2 \alpha)\Vert D_{1}x-D _{1}y \Vert ^{2} \leq \Vert x-y \Vert ^{2}. \end{aligned}$$

Thus $(I-d_{1}D_{1})$ is a nonexpansive mapping. By using the same method as above, we see that $(I-d_{2}D_{2})$ is a nonexpansive mapping. Since $f_{1}$ is a *ρ*-inverse strongly monotone mapping and $f_{2}$ is a firmly nonexpansive mapping. From Lemma 3.1(1), we have $( T^{F_{1}}_{r} ( I-rf_{1} ) ) $ and $( T^{F_{2}}_{s} ( I-sf_{2} ) ) $ are nonexpansive mappings. Since $z\in \bigcap^{N}_{i=1} F ( T_{i} ) $ and Lemma 3.3, we have

3.7 $$ \Biggl\Vert P_{C} \Biggl( I-\lambda_{n} \Biggl( \sum^{N}_{i=1}k_{i} ( I-T _{i} ) \Biggr) \Biggr) y_{n}-z \Biggr\Vert ^{2} \leq \Vert y_{n}-z \Vert ^{2}. $$

Since $z \in VI(C,D_{1})$ and $z \in VI(C,D_{2})$ and using the property of $(I-d_{1}D_{1})$ and $(I-d_{2}D_{2})$, we get

3.8 $$\begin{aligned} \Vert y_{n}-z \Vert ^{2} &= \bigl\Vert P_{C} ( I-d_{1}D_{1} ) \bigl( au_{n}+ ( 1-a ) P_{C} ( I-d_{2}D_{2} ) u_{n} \bigr) -P _{C} ( I-d_{1}D_{1} ) z \bigr\Vert ^{2} \\ &\leq a\Vert u_{n}-z\Vert ^{2}+ ( 1-a ) \bigl\Vert P_{C} ( I-d_{2}D_{2} ) u_{n}-z \bigr\Vert ^{2} \end{aligned}$$

3.9 $$\begin{aligned} &\leq \Vert u_{n}-z \Vert ^{2}. \end{aligned}$$

Since $z \in \Omega $, we have $z=T^{F_{1}}_{r} ( I-rf_{1} ) z$ and $Az=T^{F_{2}}_{s} ( I-sf_{2} ) Az$. From Lemma 3.1(2) and $\gamma \in (0,1/L)$, we obtain

3.10 $$\begin{aligned} \Vert u_{n}-z \Vert ^{2} &= \bigl\Vert T^{F_{1}}_{r} ( I-rf_{1} ) \bigl( x_{n}+ \gamma A^{*} \bigl( T^{F_{2}}_{s} ( I-sf_{2} ) -I \bigr) Ax_{n} \bigr) -T^{F_{1}}_{r} ( I-rf_{1} ) z \bigr\Vert ^{2} \\ &\leq \Vert x_{n}-z\Vert ^{2}+\gamma ( L\gamma -1 ) \bigl\Vert \bigl( T ^{F_{2}}_{s} ( I-sf_{2} ) -I \bigr) Ax_{n} \bigr\Vert ^{2} \end{aligned}$$

3.11 $$\begin{aligned} &\leq \Vert x_{n}-z\Vert ^{2}. \end{aligned}$$

Using the definition of $x_{n}$, (3.7), (3.9) and (3.11), we get

$$\begin{aligned} \Vert x_{n+1}-z\Vert = {}& \Biggl\Vert \alpha_{n}(u-z) + \beta_{n}(x_{n}-z) \\ &{}+ \gamma_{n} \Biggl( P_{C} \Biggl( I - \lambda_{n} \Biggl( \sum^{N} _{i=1}k_{i} ( I-T_{i} ) \Biggr) \Biggr) y_{n}-z \Biggr) \Biggr\Vert \\ \leq {}& \alpha_{n}\Vert u-z\Vert + \beta_{n}\Vert x_{n}-z\Vert + \gamma_{n}\Vert y_{n}-z \Vert \\ \leq {}& \alpha_{n}\Vert u-z\Vert + \beta_{n}\Vert x_{n}-z\Vert + \gamma_{n}\Vert u_{n}-z \Vert \\ \leq {}& \alpha_{n}\Vert u-z\Vert + (1-\alpha_{n}) \Vert x_{n}-z\Vert . \end{aligned}$$

Using induction, we can conclude that

$$\Vert x_{n}-z\Vert \leq \max \bigl\{ \Vert u-z\Vert , \Vert x_{1}-z\Vert \bigr\} $$

for all $n\geq 1$. This implies that the sequence $\{x_{n}\}$ is bounded and so are $\{y_{n}\}$ and $\{u_{n}\}$. From Lemma 3.1 (2) and $\gamma \in (0,1/L)$, we obtain

3.12 $$\begin{aligned} &\Vert u_{n}-u_{n-1} \Vert ^{2} \\ &\quad = \bigl\Vert T^{F_{1}}_{r} ( I-rf_{1} ) \bigl( x_{n}+\gamma A^{*} \bigl( T^{F_{2}}_{s} ( I-sf_{2} ) -I \bigr) Ax_{n} \bigr) \\ &\qquad {}- T^{F_{1}}_{r} ( I-rf_{1} ) \bigl( x_{n-1}+\gamma A^{*} \bigl( T^{F_{2}}_{s} ( I-sf_{2} ) -I \bigr) Ax_{n-1} \bigr) \bigr\Vert ^{2} \\ &\quad \leq \Vert x_{n}-x_{n-1} \Vert ^{2}+ \gamma ( \gamma L-1) \bigl\Vert \bigl( T^{F_{2}}_{s} ( I-sf_{2} ) -I \bigr) Ax_{n}- \bigl( T ^{F_{2}}_{s} ( I-sf_{2} ) -I \bigr) Ax_{n-1} \bigr\Vert ^{2} \\ &\quad \leq \Vert x_{n}-x_{n-1} \Vert ^{2}. \end{aligned}$$

Next, we show that $\lim_{n \rightarrow \infty } \Vert x_{n+1} - x_{n} \Vert =0$. According to Eq. (3.12), we have

$$\begin{aligned} &\Vert x_{n+1}-x_{n}\Vert \\ &\quad = \Biggl\Vert \Biggl( \alpha_{n}u + \beta_{n}x_{n} + \gamma_{n}P_{C} \Biggl( I - \lambda_{n} \Biggl( \sum^{N}_{i=1}k_{i} ( I-T_{i} ) \Biggr) \Biggr) y _{n} \Biggr) \\ &\qquad {}- \Biggl( \alpha_{n-1}u + \beta_{n-1}x_{n-1} + \gamma_{n-1}P _{C} \Biggl( I - \lambda_{n-1} \Biggl( \sum^{N}_{i=1}k_{i} ( I-T_{i} ) \Biggr) \Biggr) y _{n-1} \Biggr) \Biggr\Vert \\ &\quad \leq \vert \alpha_{n}-\alpha_{n-1}\vert \Vert u \Vert +\beta _{n}\Vert x_{n}-x_{n-1}\Vert + \vert \beta_{n}-\beta_{n-1}\vert \Vert x_{n-1} \Vert + \gamma_{n}\Vert y_{n}-y_{n-1}\Vert \\ &\qquad {}+\lambda_{n} \Biggl\Vert \Biggl( \sum ^{N}_{i=1}k_{i} ( I-T_{i} ) \Biggr) y _{n}- \Biggl( \sum^{N}_{i=1}k_{i} ( I-T_{i} ) \Biggr) y_{n-1} \Biggr\Vert \\ &\qquad {}+ \vert \lambda_{n}-\lambda_{n-1}\vert \Biggl\Vert \Biggl( \sum^{N}_{i=1}k _{i} ( I-T_{i} ) \Biggr) y_{n-1} \Biggr\Vert \\ &\qquad {}+ \vert \gamma_{n}-\gamma_{n-1}\vert \Biggl\Vert P_{C} \Biggl( I - \lambda_{n-1} \Biggl( \sum ^{N}_{i=1}k_{i} ( I-T_{i} ) \Biggr) \Biggr) y _{n-1} \Biggr\Vert \\ &\quad \leq (1-\alpha_{n})\Vert x_{n}-x_{n-1} \Vert +\vert \alpha_{n}- \alpha_{n-1}\vert \Vert u \Vert + \vert \beta_{n}-\beta_{n-1}\vert \Vert x_{n-1} \Vert \\ &\qquad {}+\lambda_{n} \Biggl\Vert \Biggl( \sum ^{N}_{i=1}k_{i} ( I-T_{i} ) \Biggr) y _{n}- \Biggl( \sum^{N}_{i=1}k_{i} ( I-T_{i} ) \Biggr) y_{n-1} \Biggr\Vert \\ &\qquad {}+\vert \lambda_{n}-\lambda_{n-1}\vert \Biggl\Vert \Biggl( \sum^{N}_{i=1}k _{i} ( I-T_{i} ) \Biggr) y_{n-1} \Biggr\Vert \\ &\qquad {}+ \vert \gamma_{n}-\gamma_{n-1}\vert \Biggl\Vert P_{C} \Biggl( I - \lambda_{n-1} \Biggl( \sum ^{N}_{i=1}k_{i} ( I-T_{i} ) \Biggr) \Biggr) y _{n-1} \Biggr\Vert \\ &\quad \leq (1-\alpha_{n})\Vert x_{n}-x_{n-1} \Vert +\vert \alpha_{n}- \alpha_{n-1}\vert M+\vert \beta_{n}-\beta_{n-1}\vert M+\lambda_{n}M \\ &\qquad {}+\vert \lambda_{n}-\lambda_{n-1}\vert M+ \vert \gamma_{n}-\gamma _{n-1}\vert M, \end{aligned}$$

where

$$\begin{aligned} M :=&\max_{n \in \mathbb{N}} \Biggl\{ \Vert u\Vert , \Vert x_{n}\Vert , \Biggl\Vert \Biggl( \sum ^{N}_{i=1}k_{i} ( I-T_{i} ) \Biggr) y_{n+1}- \Biggl( \sum^{N}_{i=1}k _{i} ( I-T_{i} ) \Biggr) y_{n} \Biggr\Vert , \\ &{} \Biggl\Vert \Biggl( \sum^{N}_{i=1}k_{i} ( I-T_{i} ) \Biggr) y _{n} \Biggr\Vert , \Biggl\Vert P_{C} \Biggl( I - \lambda_{n} \Biggl( \sum ^{N}_{i=1}k _{i} ( I-T_{i} ) \Biggr) \Biggr) y_{n} \Biggr\Vert \Biggr\} . \end{aligned}$$

From condition (i), (iii), (iv) and Lemma 2.6, we have

3.13 $$ \lim_{n \rightarrow \infty } \Vert x_{n+1}-x_{n} \Vert =0. $$

According to Eqs. (3.7), (3.9) and (3.10), we have

3.14 $$\begin{aligned} \Vert x_{n+1}-z\Vert ^{2}\leq {}& \alpha_{n}\Vert u-z\Vert ^{2}+\gamma_{n} \Biggl\Vert P_{C} \Biggl( I - \lambda_{n} \Biggl( \sum ^{N}_{i=1}k_{i} ( I-T_{i} ) \Biggr) \Biggr) y _{n}-z \Biggr\Vert ^{2} \\ &{}+\beta_{n}\Vert x_{n}-z\Vert ^{2}- \beta_{n}\gamma_{n} \Biggl\Vert x_{n}-P_{C} \Biggl( I - \lambda_{n} \Biggl( \sum^{N}_{i=1}k_{i} ( I-T_{i} ) \Biggr) \Biggr) y _{n} \Biggr\Vert ^{2} \\ \leq{} & \alpha_{n}\Vert u-z\Vert ^{2}+ \beta_{n}\Vert x_{n}-z\Vert ^{2}+ \gamma_{n}\Vert y_{n}-z\Vert ^{2} \\ &{}-\beta_{n}\gamma_{n} \Biggl\Vert x_{n}-P_{C} \Biggl( I - \lambda_{n} \Biggl( \sum^{N}_{i=1}k_{i} ( I-T_{i} ) \Biggr) \Biggr) y_{n} \Biggr\Vert ^{2} \end{aligned}$$

3.15 $$\begin{aligned} \leq {}& \alpha_{n}\Vert u-z\Vert ^{2}+ \beta_{n}\Vert x_{n}-z\Vert ^{2}+ \gamma_{n}\Vert u_{n}-z\Vert ^{2} \\ &{}-\beta_{n}\gamma_{n} \Biggl\Vert x_{n}-P_{C} \Biggl( I - \lambda_{n} \Biggl( \sum^{N}_{i=1}k_{i} ( I-T_{i} ) \Biggr) \Biggr) y_{n} \Biggr\Vert ^{2} \\ \leq {}& \alpha_{n}\Vert u-z\Vert ^{2}+(1- \alpha_{n})\Vert x_{n}-z\Vert ^{2} +\gamma _{n}\gamma ( L\gamma -1 ) \bigl\Vert \bigl( T^{F_{2}}_{s} ( I-sf _{2} ) -I \bigr) Ax_{n} \bigr\Vert ^{2} \\ &{}-\beta_{n}\gamma_{n} \Biggl\Vert x_{n}-P_{C} \Biggl( I - \lambda_{n} \Biggl( \sum^{N}_{i=1}k_{i} ( I-T_{i} ) \Biggr) \Biggr) y_{n} \Biggr\Vert ^{2}. \end{aligned}$$

This implies that

$$\begin{aligned} &\gamma_{n}\gamma ( 1-L\gamma ) \bigl\Vert \bigl( T^{F_{2}}_{s} ( I-sf_{2} ) -I \bigr) Ax_{n} \bigr\Vert ^{2} \\ &\quad \leq \alpha_{n}\Vert u-z\Vert ^{2}+\Vert x_{n}-x_{n+1}\Vert \bigl( \Vert x_{n}-z\Vert +\Vert x_{n+1}-z\Vert \bigr). \end{aligned}$$

By using condition (i) and (3.13), we have

3.16 $$ \lim_{n\rightarrow \infty } \bigl\Vert \bigl( T^{F_{2}}_{s} ( I-sf_{2} ) -I \bigr) Ax _{n} \bigr\Vert =0. $$

By using the same method as (3.16), we have

3.17 $$ \lim_{n\rightarrow \infty } \Biggl\Vert x_{n}-P_{C} \Biggl( I - \lambda_{n} \Biggl( \sum^{N}_{i=1}k_{i} ( I-T_{i} ) \Biggr) \Biggr) y_{n} \Biggr\Vert =0. $$

Let $M_{n} = x_{n}+\gamma A^{*} ( T^{F_{2}}_{s} ( I-sf_{2} ) -I ) Ax_{n}$. Applying the inequality (3.11), we have

3.18 $$ \Vert M_{n}-z \Vert \leq \Vert x_{n}-z\Vert . $$

Using the property of inverse strongly monotone operators and (3.18), we have

3.19 $$\begin{aligned} \Vert u_{n}-z \Vert ^{2} = {}& \bigl\Vert T^{F_{1}}_{r} ( I-rf_{1} ) M _{n} -T^{F_{1}}_{r} ( I-rf_{1} ) z \bigr\Vert ^{2} \\ \leq {}& \bigl\Vert ( I-rf_{1} ) M_{n} - ( I-rf_{1} ) z \bigr\Vert ^{2} \\ ={} & \Vert M_{n}-z \Vert ^{2}-2r\langle M_{n}-z, f_{1}M_{n}-f_{1}z \rangle +r^{2}\Vert f_{1}M_{n}-f_{1}z \Vert ^{2} \\ \leq{} & \Vert x_{n}-z \Vert ^{2}+r ( r-2\rho ) \Vert f_{1}M_{n}-f_{1}z \Vert ^{2}. \end{aligned}$$

Substituting (3.19) in (3.15), we have

$$\begin{aligned} \Vert x_{n+1}-z\Vert ^{2} \leq {}& \alpha_{n} \Vert u-z\Vert ^{2}+\beta_{n}\Vert x_{n}-z \Vert ^{2} \\ &{}+\gamma_{n} \bigl( \Vert x_{n}-z \Vert ^{2}+r ( r-2\rho ) \Vert f_{1}M_{n}-f_{1}z \Vert ^{2} \bigr) \\ \leq {}& \alpha_{n}\Vert u-z\Vert ^{2}+(1- \alpha_{n})\Vert x_{n}-z\Vert ^{2}+\gamma _{n}r ( r-2\rho ) \Vert f_{1}M_{n}-f_{1}z \Vert ^{2}. \end{aligned}$$

That is,

$$\gamma_{n}r ( 2\rho -r ) \Vert f_{1}M_{n}-f_{1}z \Vert ^{2} \leq \alpha_{n}\Vert u-z\Vert ^{2}+\Vert x_{n}-x_{n+1}\Vert \bigl( \Vert x_{n}-z\Vert +\Vert x_{n+1}-z\Vert \bigr). $$

According to condition (i) and (3.13), we get

3.20 $$ \lim_{n \rightarrow \infty } \Vert f_{1}M_{n}-f_{1}z \Vert = 0. $$

By the property of firmly nonexpansive mappings, we have

3.21 $$\begin{aligned} \Vert u_{n}-z \Vert ^{2} = {}& \bigl\Vert T^{F_{1}}_{r} ( I-rf_{1} ) M _{n} -T^{F_{1}}_{r} ( I-rf_{1} ) z \bigr\Vert ^{2} \\ \leq {}& \bigl\langle u_{n}-z,(I-rf_{1})M_{n}-(I-rf_{1})z \bigr\rangle \\ = {}& \frac{1}{2} \bigl( \Vert u_{n}-z\Vert ^{2}+ \bigl\Vert (I-rf_{1})M_{n}-(I-rf_{1})z \bigr\Vert ^{2} \\ &{}- \bigl\Vert (u_{n}-z)- \bigl( (I-rf_{1})M_{n}-(I-rf_{1})z \bigr) \bigr\Vert ^{2} \bigr). \end{aligned}$$

That is,

3.22 $$\begin{aligned} \Vert u_{n}-z \Vert ^{2} \leq {}& \bigl\Vert (I-rf_{1})M_{n}-(I-rf_{1})z \bigr\Vert ^{2}- \bigl\Vert (u_{n}-M_{n})+r ( f_{1}M_{n}-f_{1}z ) \bigr\Vert ^{2} \\ \leq {}& \Vert M_{n}-z\Vert ^{2}- \bigl( \Vert u_{n}-M_{n}\Vert ^{2}+2r\langle u_{n}-M _{n}, f_{1}M_{n}-f_{1}z \rangle \\ &{}+ r^{2}\Vert f_{1}M_{n}-f_{1}z \Vert ^{2} \bigr) \\ \leq {}& \Vert M_{n}-z\Vert ^{2}-\Vert u_{n}-M_{n}\Vert ^{2}+2r\Vert u_{n}-M_{n}\Vert \Vert f_{1}M_{n}-f_{1}z \Vert \\ &{}- r^{2}\Vert f_{1}M_{n}-f_{1}z \Vert ^{2}. \end{aligned}$$

Substituting (3.22) in (3.15), we get

$$\begin{aligned} \Vert x_{n+1}-z\Vert ^{2} \leq {}& \alpha_{n} \Vert u-z\Vert ^{2}+\beta_{n}\Vert x_{n}-z \Vert ^{2}+\gamma_{n} \bigl( \Vert M_{n}-z \Vert ^{2}-\Vert u_{n}-M_{n}\Vert ^{2} \\ &{}+ 2r\Vert u_{n}-M_{n}\Vert \Vert f_{1}M_{n}-f_{1}z\Vert -r^{2}\Vert f_{1}M_{n}-f_{1}z\Vert ^{2} \bigr) \\ \leq {}& \alpha_{n}\Vert u-z\Vert ^{2}+(1- \alpha_{n})\Vert x_{n}-z\Vert ^{2}-\gamma _{n}\Vert u_{n}-M_{n}\Vert ^{2} \\ &{}+ 2r\gamma_{n}\Vert u_{n}-M_{n}\Vert \Vert f_{1}M_{n}-f_{1}z\Vert . \end{aligned}$$

It follows that

$$\begin{aligned} \gamma_{n}\Vert u_{n}-M_{n}\Vert ^{2} \leq {}& \alpha_{n}\Vert u-z\Vert ^{2}+ \Vert x_{n}-x_{n+1}\Vert \bigl( \Vert x_{n}-z \Vert +\Vert x_{n+1}-z\Vert \bigr) \\ &{}+ 2r\gamma_{n}\Vert u_{n}-M_{n}\Vert \Vert f_{1}M_{n}-f_{1}z\Vert . \end{aligned}$$

From condition (i), (3.13) and (3.20), we ensure that

3.23 $$ \lim_{n \rightarrow \infty } \Vert u_{n}-M_{n} \Vert = 0. $$

From (3.16) and (3.23), we also have

$$\begin{aligned} \Vert u_{n}-x_{n}\Vert &\leq \Vert u_{n}-M_{n}\Vert +\Vert M_{n}-x_{n} \Vert \\ &= \Vert u_{n}-M_{n}\Vert + \bigl\Vert x_{n}+\gamma A^{*} \bigl( T^{F_{2}}_{s} ( I-sf_{2} ) -I \bigr) Ax_{n}-x_{n} \bigr\Vert \\ &\leq \Vert u_{n}-M_{n}\Vert +\gamma \Vert A\Vert \bigl\Vert \bigl( T^{F_{2}}_{s} ( I-sf_{2} ) -I \bigr) Ax_{n} \bigr\Vert . \end{aligned}$$

Then we have

3.24 $$ \lim_{n \rightarrow \infty } \Vert u_{n}-x_{n} \Vert = 0. $$

By using the same method as (3.19), we have

3.25 $$ \bigl\Vert P_{C} ( I-d_{2}D_{2} ) u_{n}-z \bigr\Vert ^{2} \leq \Vert x_{n}-z \Vert ^{2}+d _{2}(d_{2}-2\beta)\Vert D_{2}u_{n}-D_{2}z\Vert ^{2}. $$

Substituting (3.8) and (3.25) in (3.14), we have

$$\begin{aligned} \Vert x_{n+1}-z\Vert ^{2} \leq{} & \alpha_{n} \Vert u-z\Vert ^{2}+\beta_{n}\Vert x_{n}-z \Vert ^{2}+\gamma_{n} \bigl( a\Vert u_{n}-z \Vert ^{2} \\ &{}+ ( 1-a ) \bigl\Vert P_{C} ( I-d_{2}D_{2} ) u _{n}-z \bigr\Vert ^{2} \bigr) \\ \leq {}& \alpha_{n}\Vert u-z\Vert ^{2}+(1- \alpha_{n})\Vert x_{n}-z\Vert ^{2} \\ &{}+ \gamma_{n} ( 1-a ) d_{2}(d_{2}-2\beta) \Vert D_{2}u_{n}-D_{2}z\Vert ^{2}. \end{aligned}$$

We can conclude that

$$\begin{aligned} &\gamma_{n} ( 1-a )d_{2}(2\beta -d_{2})\Vert D_{2}u_{n}-D_{2}z\Vert ^{2} \\ &\quad \leq \alpha_{n}\Vert u-z\Vert ^{2}+\Vert x_{n}-x_{n+1}\Vert \bigl( \Vert x_{n}-z\Vert +\Vert x_{n+1}-z\Vert \bigr). \end{aligned}$$

According to condition (i) and (3.13), we get

3.26 $$ \lim_{n \rightarrow \infty } \Vert D_{2}u_{n}-D_{2}z \Vert = 0. $$

Since $P_{C}$ is a firmly nonexpansive mapping and using the same method as (3.21), we get

$$\begin{aligned} & \bigl\Vert P_{C} ( I-d_{2}D_{2} ) u_{n}-z \bigr\Vert ^{2} \\ &\quad \leq \frac{1}{2} \bigl( \bigl\Vert P_{C} ( I-d_{2}D_{2} ) u_{n}-z \bigr\Vert ^{2}+ \bigl\Vert ( I-d_{2}D_{2} ) u_{n}- ( I-d_{2}D_{2} ) z \bigr\Vert ^{2} \\ &\qquad {}- \bigl\Vert P_{C} ( I-d_{2}D_{2} ) u_{n}-z- ( I-d_{2}D_{2} ) u_{n}+ ( I-d_{2}D_{2} ) z \bigr\Vert ^{2} \bigr). \end{aligned}$$

That is,

3.27 $$\begin{aligned} \bigl\Vert P_{C} ( I-d_{2}D_{2} ) u_{n}-z \bigr\Vert ^{2} \leq {}& \Vert u_{n}-z \Vert ^{2}- \bigl\Vert \bigl(P_{C} ( I-d_{2}D_{2} ) u_{n}-u_{n} \bigr)+d_{2} ( D_{2}u_{n}-D_{2}z ) \bigr\Vert ^{2} \\ \leq {}& \Vert x_{n}-z\Vert ^{2}- \bigl\Vert P_{C} ( I-d_{2}D_{2} ) u_{n}-u_{n} \bigr\Vert ^{2} \\ &{}+ 2d_{2} \bigl\Vert P_{C} ( I-d_{2}D_{2} ) u_{n}-u_{n} \bigr\Vert \Vert D_{2}u_{n}-D_{2}z \Vert \\ &{}- d_{2}^{2}\Vert D_{2}u_{n}-D_{2}z \Vert ^{2}. \end{aligned}$$

Substituting (3.8) and (3.27) in (3.14), we have

$$\begin{aligned} &\Vert x_{n+1}-z\Vert ^{2} \\ &\quad \leq \alpha_{n}\Vert u-z\Vert ^{2}+ \beta_{n}\Vert x_{n}-z\Vert ^{2}+ \gamma_{n} \bigl( a\Vert u_{n}-z\Vert ^{2}+ ( 1-a ) \bigl\Vert P_{C} ( I-d_{2}D_{2} ) u_{n}-z \bigr\Vert ^{2} \bigr) \\ &\quad \leq \alpha_{n}\Vert u-z\Vert ^{2}+ \beta_{n}\Vert x_{n}-z\Vert ^{2}+ \gamma_{n} \bigl( a\Vert x_{n}-z\Vert ^{2}+ ( 1-a ) \bigl( \Vert x_{n}-z\Vert ^{2} \\ &\qquad {}- \bigl\Vert P_{C} ( I-d_{2}D_{2} ) u_{n}-u_{n} \bigr\Vert ^{2} + 2d_{2} \bigl\Vert P_{C} ( I-d_{2}D_{2} ) u_{n}-u_{n} \bigr\Vert \Vert D_{2}u_{n}-D_{2}z \Vert \\ &\qquad {}- d_{2}^{2}\Vert D_{2}u_{n}-D_{2}z \Vert ^{2} \bigr) \bigr) \\ &\quad \leq \alpha_{n}\Vert u-z\Vert ^{2}+(1- \alpha_{n})\Vert x_{n}-z\Vert ^{2}-\gamma _{n}(1-a) \bigl\Vert P_{C} ( I-d_{2}D_{2} ) u_{n}-u_{n} \bigr\Vert ^{2} \\ &\qquad {}+ 2d_{2}\gamma_{n}(1-a) \bigl\Vert P_{C} ( I-d_{2}D_{2} ) u_{n}-u_{n} \bigr\Vert \Vert D_{2}u_{n}-D_{2}z\Vert . \end{aligned}$$

Therefore

$$\begin{aligned} &\gamma_{n}(1-a) \bigl\Vert P_{C} ( I-d_{2}D_{2} ) u_{n}-u_{n} \bigr\Vert ^{2} \\ & \quad \leq \alpha_{n}\Vert u-z\Vert ^{2}+\Vert x_{n}-x_{n+1}\Vert \bigl( \Vert x_{n}-z\Vert + \Vert x_{n+1}-z\Vert \bigr) \\ &\qquad {}+ 2d_{2}\gamma_{n}(1-a) \bigl\Vert P_{C} ( I-d_{2}D_{2} ) u_{n}-u_{n} \bigr\Vert \Vert D_{2}u_{n}-D_{2}z\Vert . \end{aligned}$$

From condition (i), (3.13) and (3.26), we get

3.28 $$ \lim_{n \rightarrow \infty } \bigl\Vert P_{C} ( I-d_{2}D_{2} ) u _{n}-u_{n} \bigr\Vert = 0. $$

Let $k_{n} = au_{n}+ ( 1-a ) P_{C} ( I-d_{2}D_{2} ) u _{n}$. By using the same method as (3.19), we have

3.29 $$ \Vert y_{n}-z\Vert ^{2} \leq \Vert x_{n}-z\Vert ^{2}+d_{1}(d_{1}-2 \alpha)\Vert D_{1}k_{n}-D_{1}z\Vert ^{2}. $$

Substituting (3.29) in (3.14), we have

$$\begin{aligned} &\Vert x_{n+1}-z\Vert ^{2} \\ &\quad \leq \alpha_{n}\Vert u-z\Vert ^{2}+ \beta_{n}\Vert x_{n}-z\Vert ^{2}+ \gamma_{n} \bigl( \Vert x_{n}-z\Vert ^{2}+d_{1}(d_{1}-2\alpha)\Vert D_{1}k_{n}-D_{1}z\Vert ^{2} \bigr) \\ &\quad \leq \alpha_{n}\Vert u-z\Vert ^{2}+(1- \alpha_{n})\Vert x_{n}-z\Vert ^{2}+d_{1}(d _{1}-2\alpha)\gamma_{n}\Vert D_{1}k_{n}-D_{1}z \Vert ^{2}. \end{aligned}$$

This implies that

$$d_{1}(2\alpha -d_{1})\gamma_{n}\Vert D_{1}k_{n}-D_{1}z\Vert ^{2} \leq \alpha _{n}\Vert u-z\Vert ^{2}+\Vert x_{n}-x_{n+1}\Vert \bigl( \Vert x_{n}-z\Vert +\Vert x_{n+1}-z\Vert \bigr). $$

According to condition (i) and (3.13), we have

3.30 $$ \lim_{n \rightarrow \infty } \Vert D_{1}k_{n}-D_{1}z \Vert = 0. $$

By using the same method as (3.21), we have

$$\begin{aligned} \Vert y_{n}-z\Vert ^{2} \leq {}& \frac{1}{2} \bigl( \Vert y_{n}-z\Vert ^{2}+ \bigl\Vert ( I-d_{1}D_{1} ) k_{n}- ( I-d_{1}D_{1} ) z \bigr\Vert ^{2} \\ &{}- \bigl\Vert (y_{n}-k_{n})+d_{1}(D_{1}k_{n}-D_{1}z) \bigr\Vert ^{2} \bigr). \end{aligned}$$

That is,

3.31 $$\begin{aligned} \Vert y_{n}-z\Vert ^{2} \leq {}& \Vert k_{n}-z\Vert ^{2}- \bigl( \Vert y_{n}-k_{n} \Vert ^{2}+2d _{1}\langle y_{n}-k_{n},D_{1}k_{n}-D_{1}z \rangle \\ &{}+ d_{1}^{2}\Vert D_{1}k_{n}-D_{1}z \Vert ^{2} \bigr) \\ \leq {}& \Vert x_{n}-z\Vert ^{2}-\Vert y_{n}-k_{n}\Vert ^{2}+2d_{1}\Vert y_{n}-k_{n}\Vert \Vert D_{1}k_{n}-D_{1}z \Vert \\ &{}- d_{1}^{2}\Vert D_{1}k_{n}-D_{1}z \Vert ^{2}. \end{aligned}$$

Substituting (3.31) in (3.14), we have

3.32 $$\begin{aligned} \Vert x_{n+1}-z\Vert ^{2}\leq {}& \alpha_{n}\Vert u-z\Vert ^{2}+\beta_{n}\Vert x_{n}-z\Vert ^{2}+\gamma_{n} \bigl( \Vert x_{n}-z\Vert ^{2}-\Vert y_{n}-k_{n} \Vert ^{2} \\ &{}+ 2d_{1}\Vert y_{n}-k_{n}\Vert \Vert D_{1}k_{n}-d_{1}z\Vert -d_{1}^{2} \Vert D_{1}k_{n}-D_{1}z \Vert ^{2} \bigr) \\ \leq{} & \alpha_{n}\Vert u-z\Vert ^{2}+(1- \alpha_{n})\Vert x_{n}-z\Vert ^{2}-\gamma _{n}\Vert y_{n}-k_{n}\Vert ^{2} \\ &{}+ 2\gamma_{n}d_{1}\Vert y_{n}-k_{n} \Vert \Vert D_{1}k_{n}-D_{1}z\Vert . \end{aligned}$$

This implies that

$$\begin{aligned} \gamma_{n}\Vert y_{n}-k_{n}\Vert ^{2} \leq {}& \alpha_{n}\Vert u-z\Vert ^{2}+ \Vert x_{n}-x_{n+1}\Vert \bigl( \Vert x_{n}-z \Vert +\Vert x_{n+1}-z\Vert \bigr) \\ &{}+ 2\gamma_{n}d_{1}\Vert y_{n}-k_{n} \Vert \Vert D_{1}k_{n}-D_{1}z\Vert . \end{aligned}$$

According to condition (i), (3.13) and (3.30), we get

3.33 $$ \lim_{n \rightarrow \infty } \Vert y_{n}-k_{n} \Vert = 0. $$

From (3.28) and (3.33)

$$\begin{aligned} \Vert y_{n}-u_{n}\Vert \leq {}& \Vert y_{n}-k_{n}\Vert +\Vert k_{n}-u_{n} \Vert \\ \leq {}& \Vert y_{n}-k_{n}\Vert +(1-a) \bigl\Vert P_{C} ( I-d_{2}D_{2} ) u_{n}-u_{n} \bigr\Vert , \end{aligned}$$

we conclude that

3.34 $$ \lim_{n \rightarrow \infty } \Vert y_{n}-u_{n} \Vert = 0. $$

By (3.24) and (3.34), we also conclude that

3.35 $$ \lim_{n \rightarrow \infty } \Vert y_{n}-x_{n} \Vert = 0. $$

Afterward, we show that $\limsup_{n \rightarrow \infty } \langle u-z,x_{n}-z \rangle \leq 0$, where $z=P_{\mathcal{F}}u$.

To show this, choose a subsequence $\{ x_{n_{j}} \} $ of $\{ x_{n} \} $ such that

3.36 $$ \limsup_{n \rightarrow \infty } \langle u-z, x_{n} - z \rangle = \lim_{j \rightarrow \infty } \langle u-z, x_{n _{j}} - z \rangle. $$

Without loss of generality, we may assume that $x_{n_{j}} \rightharpoonup \omega $ as $j \rightarrow \infty $. From (3.35), we obtain $y_{n_{j}} \rightharpoonup \omega $ as $j \rightarrow \infty $. From Lemma 2.3, we have $VI ( C,D_{1} ) = F ( P_{C}(I-d _{1}D_{1}) ) $. Assume that $\omega \notin VI ( C,D_{1} ) $, we have $\omega \neq P_{C}(I-d _{1}D_{1})\omega $. Using Opial’s condition, (3.33), we obtain

$$\begin{aligned} \liminf_{j \rightarrow \infty } \Vert y_{n_{j}} - \omega \Vert < {}& \liminf_{j \rightarrow \infty } \bigl\Vert y_{n_{j}} - P_{C}(I-d_{1}D _{1})\omega \bigr\Vert \\ \leq{} & \liminf_{j \rightarrow \infty } \bigl( \bigl\Vert P_{C}(I-d_{1}D _{1})k_{n_{j}} - P_{C}(I-d_{1}D_{1})y_{n_{j}} \bigr\Vert \\ &{}+ \bigl\Vert P_{C}(I-d_{1}D_{1})y_{n_{j}} - P_{C}(I-d_{1}D_{1}) \omega \bigr\Vert \bigr) \\ \leq {}& \liminf_{j \rightarrow \infty } \bigl( \Vert k_{n_{j}} - y _{n_{j}}\Vert + \Vert y_{n_{j}} - \omega \Vert \bigr) \\ \leq{} & \liminf_{j \rightarrow \infty } \Vert y_{n_{j}} - \omega \Vert . \end{aligned}$$

This is a contradiction, so we have

3.37 $$ \omega \in VI ( C,D_{1} ). $$

From (3.24), we have $u_{n_{j}} \rightharpoonup \omega $ as $j \rightarrow \infty $. By (3.28) and using the same method as (3.37), we obtain

3.38 $$ \omega \in VI ( C,D_{2} ). $$

Next, we show that $\omega \in \bigcap^{N}_{i=1} F ( T_{i} ) $. From Lemma 2.5, we have

$$\bigcap^{N}_{i=1} F ( T_{i} ) = F \Biggl( P_{C} \Biggl( I - \lambda_{n_{j}} \Biggl( \sum ^{N}_{i=1}k_{i} ( I-T_{i} ) \Biggr) \Biggr) \Biggr). $$

Assume that $\omega \notin \bigcap^{N}_{i=1} F ( T_{i} ) $, and that $\omega \neq P_{C} ( I - \lambda_{n_{j}} ( \sum^{N}_{i=1}k _{i} ( I-T_{i} ) ) ) \omega $. Using Opial’s condition, (3.17) and (3.35), we obtain

$$\begin{aligned} & \liminf_{j \rightarrow \infty } \Vert x_{n_{j}} - \omega \Vert \\ &\quad < \liminf_{j \rightarrow \infty } \Biggl\Vert x_{n_{j}} - P_{C} \Biggl( I - \lambda_{n_{j}} \Biggl( \sum ^{N}_{i=1}k_{i} ( I-T_{i} ) \Biggr) \Biggr) \omega \Biggr\Vert \\ &\quad \leq \liminf_{j \rightarrow \infty } \Biggl( \Biggl\Vert x_{n_{j}} - P _{C} \Biggl( I - \lambda_{n_{j}} \Biggl( \sum^{N}_{i=1}k_{i} ( I-T_{i} ) \Biggr) \Biggr) y _{n_{j}} \Biggr\Vert \\ &\qquad {}+ \Biggl\Vert P_{C} \Biggl( I - \lambda_{n_{j}} \Biggl( \sum^{N}_{i=1}k_{i} ( I-T_{i} ) \Biggr) \Biggr) y_{n_{j}} - P_{C} \Biggl( I - \lambda_{n_{j}} \Biggl( \sum^{N}_{i=1}k_{i} ( I-T_{i} ) \Biggr) \Biggr) x _{n_{j}} \Biggr\Vert \\ &\qquad {}+ \Biggl\Vert P_{C} \Biggl( I - \lambda_{n_{j}} \Biggl( \sum^{N}_{i=1}k _{i} ( I-T_{i} ) \Biggr) \Biggr) x_{n_{j}} - P_{C} \Biggl( I - \lambda_{n_{j}} \Biggl( \sum^{N}_{i=1}k_{i} ( I-T_{i} ) \Biggr) \Biggr) \omega \Biggr\Vert \Biggr) \\ &\quad \leq \liminf_{j \rightarrow \infty } \Biggl( \Vert y_{n_{j}}-x _{n_{j}} \Vert +\lambda_{n_{j}} \Biggl\Vert \Biggl( \sum ^{N}_{i=1}k_{i} ( I-T_{i} ) \Biggr) y_{n_{j}} - \Biggl( \sum ^{N}_{i=1}k_{i} ( I-T_{i} ) \Biggr) x_{n_{j}} \Biggr\Vert \\ &\qquad {}+ \Vert x_{n_{j}} - \omega \Vert +\lambda_{n_{j}} \Biggl\Vert \Biggl( \sum^{N}_{i=1}k_{i} ( I-T_{i} ) \Biggr) x_{n_{j}}- \Biggl( \sum ^{N}_{i=1}k_{i} ( I-T_{i} ) \Biggr) \omega \Biggr\Vert \Biggr) \\ &\quad \leq \liminf_{j \rightarrow \infty } \Vert x_{n_{j}} - \omega \Vert . \end{aligned}$$

This is a contradiction, so we have

3.39 $$ \omega \in \bigcap^{N}_{i=1} F ( T_{i} ). $$

After that, we show that $\omega \in \Omega $. Assume $\omega \notin EP(F_{1},f_{1})$. Since $EP(F_{1},f_{1})=F(T^{F_{1}}_{r} ( I-rf _{1} ))$, we obtain $\omega \neq T^{F_{1}}_{r} ( I-rf_{1} ) \omega $. Using Opial’s condition and (3.23), we get

$$\begin{aligned} \liminf_{j \rightarrow \infty } \Vert u_{n_{j}} - \omega \Vert < {}& \liminf_{j \rightarrow \infty } \bigl\Vert u_{n_{j}} - T^{F_{1}}_{r} ( I-rf_{1} ) \omega \bigr\Vert \\ \leq {}& \liminf_{j \rightarrow \infty } \bigl( \bigl\Vert T^{F_{1}}_{r} ( I-rf_{1} ) M_{n_{j}} - T^{F_{1}}_{r} ( I-rf_{1} ) u _{n_{j}} \bigr\Vert \\ &{}+ \bigl\Vert T^{F_{1}}_{r} ( I-rf_{1} ) u_{n_{j}} - T ^{F_{1}}_{r} ( I-rf_{1} ) \omega \bigr\Vert \bigr) \\ \leq {}& \liminf_{j \rightarrow \infty } \bigl( \Vert M_{n_{j}} - u _{n_{j}}\Vert +\Vert u_{n_{j}} - \omega \Vert \bigr) \\ \leq {}& \liminf_{j \rightarrow \infty } \Vert u_{n_{j}} - \omega \Vert . \end{aligned}$$

This is a contradiction, so we have

3.40 $$ \omega \in EP(F_{1},f_{1}). $$

Next, we show that $A\omega \in EP(F_{2},f_{2})$. Since *A* is bounded linear operator so that $Ax_{n_{j}} \rightharpoonup A\omega $ as $j\rightarrow \infty $. Assume $A\omega \notin EP(F_{2},f_{2})$. Since $EP(F_{2},f_{2})=F(T^{F_{2}}_{s} ( I-sf_{2} ))$, we obtain $A\omega \neq T^{F_{2}}_{s} ( I-sf_{s} ) A\omega $. Using Opial’s condition and (3.16), we have

3.41 $$ A\omega \in EP(F_{2},f_{2}). $$

We can conclude that $\omega \in \Omega $. Therefore $\omega \in \mathcal{F}$. Since $x_{n_{j}} \rightharpoonup \omega $ as $j\rightarrow \infty $, we have

3.42 $$\begin{aligned} \limsup_{n \rightarrow \infty } \langle u-z, x_{n} - z \rangle &= \lim_{j \rightarrow \infty } \langle u-z, x_{n _{j}} - z \rangle \\ &= \langle u-z, \omega - z \rangle \leq 0. \end{aligned}$$

Finally, we show that the sequence $\{x_{n}\}$ converges strongly to $z=P_{\mathcal{F}}u$. By (3.7), (3.9) and (3.11), we get

$$\begin{aligned} \Vert x_{n+1}-z\Vert ^{2} = {}& \Biggl\Vert \alpha_{n}(u-z) + \beta_{n}(x_{n}-z) + \gamma_{n} \Biggl( P_{C} \Biggl( I - \lambda_{n} \Biggl( \sum^{N}_{i=1}k_{i} ( I-T_{i} ) \Biggr) \Biggr) y_{n}-z \Biggr) \Biggr\Vert ^{2} \\ \leq {}& \Biggl\Vert \beta_{n}(x_{n}-z) + \gamma_{n} \Biggl( P_{C} \Biggl( I - \lambda_{n} \Biggl( \sum^{N}_{i=1}k_{i} ( I-T_{i} ) \Biggr) \Biggr) y _{n}-z \Biggr) \Biggr\Vert ^{2} \\ &{}+2\alpha_{n} \langle u-z, x_{n+1}-z \rangle \\ \leq{} & \bigl( \beta_{n}\Vert x_{n}-z\Vert + \gamma_{n}\Vert u _{n}-z\Vert \bigr) ^{2}+2 \alpha_{n} \langle u-z, x_{n+1}-z \rangle \\ \leq {}& (1-\alpha_{n})\Vert x_{n}-z\Vert ^{2}+2\alpha_{n} \langle u-z, x _{n+1}-z \rangle. \end{aligned}$$

According to condition (i), (3.42) and Lemma 2.6, we can conclude that $\{x_{n}\}$ converges strongly to $z=P_{\mathcal{F}}u$. By (3.24) and (3.35), we have $\{u_{n}\}$ and $\{y_{n}\}$ converge strongly to $z=P_{\mathcal{F}}u$. This completes the proof. □

These results are directly proved from Theorem 3.4. Therefore, we omit the proof.

### Corollary 3.5

*Let* *C* *and* *Q* *be nonempty closed convex subsets of a real Hilbert space* $H_{1}$ *and* $H_{2}$, *respectively*. *Let* $A:H_{1} \rightarrow H _{2}$ *be a bounded linear operator*. *Let* $D_{1}, D_{2}:C \rightarrow H _{1}$ *be* *α*, *β*-*inverse strongly monotone mappings*, *respectively*. *Let* $F_{1}:C \times C \rightarrow \mathbb{R}$ *and* $F_{2}:Q \times Q \rightarrow \mathbb{R}$ *be the bifunctions satisfying* (A1)*–*(A4). *Let* *T* *be a quasi*-*nonexpansive mapping of* *C* *into itself*. *Let* $f_{1}: H_{1} \rightarrow H_{1}$ *be a* *ρ*-*inverse strongly monotone mapping and* $f_{2}: H_{2} \rightarrow H_{2}$ *be a firmly nonexpansive mapping*. *Assume* $\mathcal{F}= VI(C,D_{1}) \cap VI(C,D _{2}) \cap F ( T ) \cap \Omega \neq \emptyset $. *For given* $x_{1}$, $u\in C$, *and let* $\{ x_{n} \}$, $\{ u_{n} \}$ *and* $\{ y_{n} \}$ *be sequences generated by*

$$\textstyle\begin{cases} u_{n} = T^{F_{1}}_{r} ( I-rf_{1} ) ( x_{n}+\gamma A ^{*} ( T^{F_{2}}_{s} ( I-sf_{2} ) -I ) Ax_{n} ), \\ y_{n} = P_{C} ( I-d_{1}D_{1} ) ( au_{n}+ ( 1-a ) P_{C} ( I-d_{2}D_{2} ) u_{n} ), \\ x_{n+1} = \alpha_{n}u + \beta_{n}x_{n} + \gamma_{n}P_{C} ( I - \lambda_{n} ( I-T ) ) y_{n}, \quad \forall n\in \mathbb{N}, \end{cases} $$

*where* $d_{1} \in (0,2\alpha)$, $d_{2} \in (0,2\beta)$, $r \in (0,2 \rho)$, $s \in (0,1)$, $a \in [0,1]$, $\gamma \in (0,1/L)$, *L* *is the spectral radius of the operator* $A^{*}A$ *and* $A^{*}$ *is the adjoint of* *A*. *Also* $\{ \alpha_{n} \}$, $\{ \beta_{n} \}$, $\{ \gamma_{n} \}$ *are sequences in* $[0,1]$ *with* $\alpha_{n} + \beta_{n} + \gamma_{n} = 1$ *for all* $n \in \mathbb{N}$. *Suppose the conditions* (i)*–*(iv) *of Theorem *3.4 *hold*. *Then* $\{ x_{n} \}$, $\{ u_{n} \}$ *and* $\{ y_{n} \}$ *converge strongly to* $z=P_{\mathcal{F}}u$.

### Corollary 3.6

*Let* *C* *be nonempty closed convex subset of a real Hilbert space* $H_{1}$. *Let* $D_{1}, D_{2}:C \rightarrow H_{1}$ *be* *α*, *β*-*inverse strongly monotone mappings*, *respectively*. *Let* $F_{1}:C \times C \rightarrow \mathbb{R}$ *be the bifunction satisfying* (A1)*–*(A4). *Let* $\{ T_{i} \} ^{N}_{i=1}$ *be a finite family of quasi*-*nonexpansive mappings of* *C* *into itself with* $\bigcap^{N}_{i=1} F ( T_{i} ) \neq \emptyset $. *Let* $f_{1}: H_{1} \rightarrow H_{1}$ *be a* *ρ*-*inverse strongly monotone mapping*. *Assume* $\mathcal{F}= VI(C,D_{1}) \cap VI(C,D_{2}) \cap \bigcap^{N}_{i=1} F ( T_{i} ) \cap EP(F_{1}, f_{1}) \neq \emptyset$. *For given* $x_{1},u\in C$ *and let* $\{ x_{n} \}, \{ u_{n} \}$ *and* $\{ y_{n} \}$ *be sequences generated by*

$$\textstyle\begin{cases} u_{n} = T^{F_{1}}_{r} ( I-rf_{1} ) x_{n}, \\ y_{n} = P_{C} ( I-d_{1}D_{1} ) ( au_{n}+ ( 1-a ) P_{C} ( I-d_{2}D_{2} ) u_{n} ), \\ x_{n+1} = \alpha_{n}u + \beta_{n}x_{n} + \gamma_{n}P_{C} ( I - \lambda_{n} ( \sum^{N}_{i=1}k_{i} ( I-T_{i} ) ) ) y _{n}, \quad \forall n\in \mathbb{N}, \end{cases} $$

*where* $d_{1} \in (0,2\alpha)$, $d_{2} \in (0,2\beta)$, $r \in (0,2 \rho)$, $a \in [0,1]$, $0 < k_{i} < 1$ *with* $\sum^{N}_{i=1}k_{i}=1$. *Also* $\{ \alpha_{n} \}$, $\{ \beta_{n} \}$, $\{ \gamma_{n} \}$ *are sequences in* $[0,1]$ *with* $\alpha_{n} + \beta_{n} + \gamma_{n} = 1$ *for all* $n \in \mathbb{N}$. *Suppose the conditions* (i)*–*(iv) *of Theorem *3.4 *hold*. *Then* $\{ x_{n} \}$, $\{ u_{n} \}$ *and* $\{ y_{n} \}$ *converge strongly to* $z=P_{\mathcal{F}}u$.

### Corollary 3.7

*Let* *C* *and* *Q* *be nonempty closed convex subsets of a real Hilbert space* $H_{1}$ *and* $H_{2}$, *respectively*. *Let* $A:H_{1} \rightarrow H _{2}$ *be a bounded linear operator*. *Let* $D_{1}, D_{2}:C \rightarrow H _{1}$ *be* *α*, *β*-*inverse strongly monotone mappings*, *respectively*. *Let* $F_{1}:C \times C \rightarrow \mathbb{R}$ *and* $F_{2}:Q \times Q \rightarrow \mathbb{R}$ *be the bifunctions satisfying* (A1)*–*(A4). *Let* $\{ T_{i} \} ^{N}_{i=1}$ *be a finite family of quasi*-*nonexpansive mappings of* *C* *into itself with* $\bigcap^{N}_{i=1} F ( T_{i} ) \neq \emptyset $. *Assume* $\mathcal{F}= VI(C,D_{1}) \cap VI(C,D_{2}) \cap \bigcap^{N}_{i=1} F ( T_{i} ) \cap \Gamma \neq \emptyset $. *For given* $x_{1},u \in C$ *and let* $\{ x_{n} \}$, $\{ u_{n} \}$ *and* $\{ y_{n} \}$ *be sequences generated by*

$$\textstyle\begin{cases} u_{n} = T^{F_{1}}_{r} ( x_{n}+\gamma A^{*} ( T^{F_{2}}_{s}-I ) Ax_{n} ), \\ y_{n} = P_{C} ( I-d_{1}D_{1} ) ( au_{n}+ ( 1-a ) P_{C} ( I-d_{2}D_{2} ) u_{n} ), \\ x_{n+1} = \alpha_{n}u + \beta_{n}x_{n} + \gamma_{n}P_{C} ( I - \lambda_{n} ( \sum^{N}_{i=1}k_{i} ( I-T_{i} ) ) ) y _{n},\quad \forall n\in \mathbb{N}, \end{cases} $$

*where* $d_{1} \in (0,2\alpha)$, $d_{2} \in (0,2\beta)$, $a \in [0,1]$, $0 < k_{i} < 1$ *with* $\sum^{N}_{i=1}k_{i}=1$, $\gamma \in (0,1/L)$, *L* *is the spectral radius of the operator* $A^{*}A$ *and* $A^{*}$ *is the adjoint of* *A*. *Also* $\{ \alpha_{n} \}$, $\{ \beta_{n} \}$, $\{ \gamma_{n} \}$ *are sequences in* $[0,1]$ *with* $\alpha_{n} + \beta_{n} + \gamma_{n} = 1$ *for all* $n \in \mathbb{N}$. *Suppose the conditions* (i)*–*(iv) *of Theorem *3.4 *hold*. *Then* $\{ x_{n} \}$, $\{ u_{n} \}$ *and* $\{ y_{n} \}$ *converge strongly to* $z=P_{\mathcal{F}}u$.

### Remark 3.8

If we take $N=1$ in Theorem 3.4, we have a strong convergence for finding a common element of the set of solutions of variational inequality problems and the set of fixed points of a quasi-nonexpansive mapping and the set of solutions of the modified split generalized equilibrium problem. From previous result, we can apply by using the same method as Theorem 4.5 in [24]. We have a strong convergence for finding a common element of the set of solutions of variational inequality problems and the set of fixed points of a finite family of nonspreading mappings and the set of solutions of the modified split generalized equilibrium problem. By using our main result, Theorem 3.4 reduces to the Corollary 3.6, the solution of the generalized equilibrium problem and Corollary 3.7, the split equilibrium problem. All theorems are found as regards the solution of common fixed points of a finite family of quasi-nonexpansive mappings without assuming $T_{\omega }:= (1-\omega)I+\omega T$ and *T* is demiclosed; a difficult proof in a framework of Hilbert space.

## Application

The following knowledge is used to prove Theorem 4.4. A mapping $T: C \rightarrow C$ is called nonspreading if

4.1 $$ 2\Vert Tx-Ty\Vert ^{2}\leq \Vert Tx-y\Vert ^{2}+\Vert Ty-x\Vert ^{2},\quad \forall x,y\in C. $$

Such a mapping is defined by Kohsaka and Takahashi [25].

In 2009, Iemoto and Takahashi [26] proved that (4.1) is equivalent to

4.2 $$ \Vert Tx-Ty\Vert ^{2}\leq \Vert x-y\Vert ^{2}+2\langle x-Tx,y-Ty \rangle,\quad \forall x,y \in C. $$

### Remark 4.1

A nonspreading mapping *T* with $F(T)\neq \emptyset $ is quasi-nonexpansive mapping *T*.

### Lemma 4.2

([25])

*Let* *H* *be a Hilbert space*, *let* *C* *be a nonempty closed convex subset of* *H*, *and let* *S* *be a nonspreading mapping of C into itself*. *Then* $F(S)$ *is closed and convex*.

In 2009, Kangtunyakarn and Suantai[27] introduced the *S*-mapping generated by $T_{1},T_{2},T_{3},\ldots,T_{N}$ and $\lambda _{1},\lambda_{2},\ldots,\lambda_{N}$ as follows.

### Definition 4.1

Let *C* be a nonempty convex subset of a real Banach space. Let $\{T_{i}\}_{i=1}^{N}$ be a finite family of (nonexpansive) mappings of *C* into itself. For each $j=1,2,\ldots,N$, let $\alpha_{j}=(\alpha_{1} ^{j}, \alpha_{2}^{j}, \alpha_{3}^{j})\in I\times I\times I$, where $I\in [0,1]$ and $\alpha_{1}^{j}+\alpha_{2}^{j}+\alpha_{3}^{j}=1$. Define the mapping $S:C\rightarrow C$ as follows:

$$\begin{aligned} &U_{0}=I, \\ &U_{1}=\alpha_{1}^{1}T_{1}U_{0}+ \alpha_{2}^{1}U_{0}+\alpha_{3}^{1}I, \\ &U_{2}=\alpha_{1}^{2}T_{2}U_{1}+ \alpha_{2}^{2}U_{1}+\alpha_{3}^{2}I, \\ &U_{3}=\alpha_{1}^{3}T_{3}U_{2}+ \alpha_{2}^{3}U_{2}+\alpha_{3}^{3}I, \\ &\vdots \\ &U_{N-1}=\alpha_{1}^{N-1}T_{N-1}U_{N-2}+ \alpha_{2}^{N-1}U_{N-2}+ \alpha_{3}^{N-1}I, \\ &S=U_{N}=\alpha_{1}^{N}T_{N}U_{N-1}+ \alpha_{2}^{N}U_{N-1}+\alpha_{3} ^{N}I. \end{aligned}$$

This mapping is called an *S-mapping* generated by $T_{1}, T_{2}, \ldots, T_{N}$ and $\alpha_{1}, \alpha_{2}, \ldots, \alpha_{N}$.

### Lemma 4.3

([28])

*Let* *C* *be a nonempty closed convex subset of a real Hilbert space*. *Let* $\{T_{i}\}_{i=1}^{N}$ *be a finite family of nonspreading mappings of* *C* *into* *C* *with* $\bigcap_{i=1}^{N}F(T_{i}) \neq \emptyset $, *and let* $\alpha_{j}=(\alpha_{1}^{j}, \alpha_{2}^{j}, \alpha_{3}^{j})\in I\times I\times I$, $j=1, 2, \ldots, N$, *where* $I=[0,1]$, $\alpha_{1}^{j}+\alpha_{2}^{j}+\alpha_{3}^{j}=1$, $\alpha_{1} ^{j}, \alpha_{3}^{j}\in (0,1)$ *for all* $j=1, 2, \ldots, N-1$ *and* $\alpha_{1}^{N}\in (0,1]$, $\alpha_{3}^{N}\in [0,1)$, $\alpha_{2}^{j} \in [0,1)$ *for all* $j=1, 2, \ldots, N$. *Let* *S* *be the mapping generated by* $T_{1}, T_{2}, \ldots, T_{N}$ *and* $\alpha_{1}, \alpha_{2}, \ldots, \alpha _{N}$. *Then* $F(S)=\bigcap_{i=1}^{N}F(T_{i})$ *and* *S* *is a quasi*-*nonexpansive mapping*.

By using these results, we obtain the following theorems.

### Theorem 4.4

*Let* *C* *and* *Q* *be nonempty closed convex subsets of a real Hilbert space* $H_{1}$ *and* $H_{2}$, *respectively*. *Let* $A:H_{1} \rightarrow H _{2}$ *be a bounded linear operator*. *Let* $D_{1}, D_{2}:C \rightarrow H _{1}$ *be* *α*, *β*-*inverse strongly monotone mappings*, *respectively*. *Let* $F_{1}:C \times C \rightarrow \mathbb{R}$ *and* $F_{2}:Q \times Q \rightarrow \mathbb{R}$ *be the bifunctions satisfying* (A1)*–*(A4). *Let* $\{T_{i}\}_{i=1}^{N}$ *be a finite family of nonspreading mappings of* *C* *into* *C* *with* $\bigcap_{i=1}^{N}F(T_{i})\neq \emptyset $, *and let* $\alpha_{j}=(\alpha_{1}^{j}, \alpha_{2}^{j}, \alpha_{3} ^{j})\in I\times I\times I$, $j=1, 2, \ldots, N$, *where* $I=[0,1], \alpha _{1}^{j}+\alpha_{2}^{j}+\alpha_{3}^{j}=1$, $\alpha_{1}^{j}, \alpha_{3} ^{j}\in (0,1)$ *for all* $j=1, 2, \ldots, N-1$ *and* $\alpha_{1}^{N}\in (0,1]$, $\alpha_{3}^{N}\in [0,1)$, $\alpha_{2}^{j}\in [0,1)$ *for all* $j=1, 2, \ldots, N$. *Let* *S* *be the mapping generated by* $T_{1}, T_{2}, \ldots, T_{N}$ *and* $\alpha_{1}, \alpha_{2}, \ldots, \alpha_{N}$. *Let* $f_{1}: H_{1} \rightarrow H_{1}$ *be a* *ρ*-*inverse strongly monotone mapping and* $f_{2}: H _{2} \rightarrow H_{2}$ *be a firmly nonexpansive mapping*. *Assume* $\mathcal{F}= VI(C,D_{1}) \cap VI(C,D_{2}) \cap \bigcap^{N}_{i=1} F ( T_{i} ) \cap \Omega \neq \emptyset $. *For given* $x_{1},u \in C$ *and let* $\{ x_{n} \}$, $\{ u_{n} \}$ *and* $\{ y_{n} \}$ *be sequences generated by*

$$\textstyle\begin{cases} u_{n} = T^{F_{1}}_{r} ( I-rf_{1} ) ( x_{n}+\gamma A ^{*} ( T^{F_{2}}_{s} ( I-sf_{2} ) -I ) Ax_{n} ), \\ y_{n} = P_{C} ( I-d_{1}D_{1} ) ( au_{n}+ ( 1-a ) P_{C} ( I-d_{2}D_{2} ) u_{n} ), \\ x_{n+1} = \alpha_{n}u + \beta_{n}x_{n} + \gamma_{n}P_{C} ( I - \lambda_{n} ( I-S ) ) y_{n},\quad \forall n\in \mathbb{N}, \end{cases} $$

*where* $d_{1} \in (0,2\alpha)$, $d_{2} \in (0,2\beta)$, $r \in (0,2 \rho)$, $s \in (0,1)$, $a \in [0,1]$, $\gamma \in (0,1/L)$, *L* *is the spectral radius of the operator* $A^{*}A$ *and* $A^{*}$ *is the adjoint of* *A*. *Also* $\{ \alpha_{n} \}$, $\{ \beta_{n} \}$, $\{ \gamma_{n} \}$ *are sequences in* $[0,1]$ *with* $\alpha_{n} + \beta_{n} + \gamma_{n} = 1$ *for all* $n \in \mathbb{N}$. *Suppose the conditions* (i)*–*(iv) *of Theorem *3.4 *hold*. *Then* $\{ x_{n} \}$, $\{ u_{n} \}$ *and* $\{ y_{n} \}$ *converge strongly to* $z=P_{\mathcal{F}}u$.

### Proof

By using Corollary 3.5 and Lemma 4.3, we obtain the conclusion. □

## Example and numerical results

In this section, an example is given for supporting Theorem 3.4. In Example 5.1, we only instance an example in infinite dimensional Hilbert space for supporting Theorem 3.4. We omit the computer programming.

### Example 5.1

Let $H_{1} = H_{2} = C = Q = \ell_{2}$ be the linear space whose elements consist of all 2-summable sequences $(x_{1}, x_{2}, \ldots, x _{j}, \ldots)$ of scalars, i.e.,

$$\ell_{2}= \Biggl\{ x:x=(x_{1}, x_{2}, \ldots, x_{j}, \ldots) \text{ and } \sum^{\infty }_{j=1} \vert x_{j}\vert ^{2} < \infty \Biggr\} , $$

with an inner product $\langle \cdot, \cdot \rangle: \ell _{2} \times \ell_{2} \rightarrow \mathbb{R}$ defined by $\langle x, y \rangle = \sum^{\infty }_{j=1}x_{j}y_{j}$ where $x=\{x_{j}\} ^{\infty }_{j=1}$, $y=\{y_{j}\}^{\infty }_{j=1} \in \ell_{2}$ and a norm $\Vert \cdot \Vert : \ell_{2} \rightarrow \mathbb{R}$ defined by $\Vert x \Vert _{2} = ( \sum^{\infty }_{j=1}|x_{j}|^{2} ) ^{\frac{1}{2}}$ where $x=\{x_{j}\}^{\infty }_{j=1} \in \ell_{2}$. Let the mapping $A : \ell_{2} \rightarrow \ell_{2}$ be defined by $Ax = ( \frac{x_{1}}{3}, \frac{x_{2}}{3}, \ldots, \frac{x_{j}}{3}, \ldots ) $ for all $x=\{x_{j}\}^{\infty }_{j=1} \in \ell_{2}$ and $A^{*} : \ell_{2} \rightarrow \ell_{2}$ be defined by $A^{*}z = ( \frac{z _{1}}{3}, \frac{z_{2}}{3}, \ldots, \frac{z_{j}}{3}, \ldots ) $ for all $z=\{z_{j}\}^{\infty }_{j=1} \in \ell_{2}$. Let $D_{1},D_{2} : \ell _{2} \rightarrow \ell_{2}$ be defined by $D_{1}x = ( \frac{x_{1}}{6}, \frac{x_{2}}{6}, \ldots, \frac{x_{j}}{6}, \ldots ) $ and $D_{2}x = ( \frac{x_{1}}{5}, \frac{x_{2}}{5}, \ldots, \frac{x_{j}}{5}, \ldots )$, $\forall x=\{x_{j}\}^{\infty }_{j=1} \in \ell_{2}$, respectively. Let the mapping $T_{i}: \ell_{2} \rightarrow \ell_{2}$ be defined by $T_{i}x = ( \frac{3ix_{1}}{5i+1}, \frac{3ix _{2}}{5i+1}, \ldots, \frac{3ix_{j}}{5i+1}, \ldots )$, $\forall x=\{x_{j} \}^{\infty }_{j=1} \in \ell_{2}$ and $k_{i} = \frac{6}{7^{i}} + \frac{1}{N7^{N}}$ for every $i = 1, 2, \ldots, N$. Let the mapping $F_{1}, F_{2} : \mathbb{R}^{2}\times \mathbb{R}^{2} \rightarrow \mathbb{R}$ be defined by

$$F_{1}(x, y) = -x^{2}+y^{2},\quad \forall x= \{x_{j}\}^{\infty }_{j=1}, y=\{y_{j} \}^{\infty }_{j=1} \in \ell_{2}, $$

and

$$F_{2}(x, y) = -2x^{2}+xy+y^{2},\quad \forall x= \{x_{j}\}^{\infty }_{j=1}, y=\{y_{j} \}^{\infty }_{j=1} \in \ell_{2}. $$

Let the mapping $f_{1}: \ell_{2} \rightarrow \ell_{2}$ be defined by $f_{1}x = ( \frac{x_{1}}{5}, \frac{x_{2}}{5}, \ldots, \frac{x_{j}}{5}, \ldots )$, $\forall x=\{x_{j}\}^{\infty }_{j=1} \in \ell_{2}$ and the mapping $f_{2}: \ell_{2} \rightarrow \ell_{2}$ be defined by $f_{2}x = ( \frac{x_{1}}{7}, \frac{x_{2}}{7}, \ldots, \frac{x _{j}}{7}, \ldots )$, $\forall x=\{x_{j}\}^{\infty }_{j=1} \in \ell _{2}$. Let $r=1$ and $s=0.5$. Since $L=\frac{1}{9}$, we choose $\gamma =0.5$. Let $x_{1}=(x_{1}^{1}, x_{1}^{2}, \ldots, x_{1}^{j}, \ldots)$ and $u=(u_{1}, u_{2}, \ldots, u_{j}, \ldots)\in \ell_{2}$ and let the sequences $\{ x_{n} \} $, $\{ y_{n} \} $ and $\{ u _{n} \} $ be generated by (3.6) as follows:

$$\textstyle\begin{cases} \textstyle\begin{array}{l l l} u_{n} = T^{F_{1}}_{1} ( I-f_{1} ) ( x_{n}+0.5A^{*} ( T^{F_{2}}_{0.5} ( I-0.5f_{2} ) -I ) Ax_{n} ), \\ y_{n} = ( I-D_{1} ) ( 0.5u_{n}+0.5 ( I-D_{2} ) u _{n} ), \\ x_{n+1} = \frac{1}{2n}u + \frac{7n-4}{12n}x_{n} + \frac{5n-2}{12n} ( y_{n} - ( (\frac{1}{2n^{2}}) ( \sum^{N}_{i=1}( \frac{6}{7^{i}}+\frac{1}{N7^{N}}) ( y_{n}-T_{i}y_{n} ) ) ) ), \end{array}\displaystyle \end{cases} $$

for all $n\geq 1$, where $x_{n}=(x_{n}^{1}, x_{n}^{2}, \ldots, x_{n}^{j}, \ldots), y_{n}=(y_{n}^{1}, y_{n}^{2}, \ldots, y_{n}^{j}, \ldots)$ and $u_{n}=(u_{n}^{1}, u_{n}^{2}, \ldots, u_{n}^{j}, \ldots)$. It easy to see that $D_{1}$, $D_{2}$, $T_{i}$, $F_{1}$, $F_{2}$, $f_{1}$ and $f_{2}$ satisfy Theorem 3.4. Moreover, we have $VI(C,D_{1}) \cap VI(C,D_{2}) \cap \bigcap^{N}_{i=1} F ( T_{i} ) \cap \Omega =\{0\}$, where $\rho =d_{1}=d_{2}=1$. From Theorem 3.4, we can conclude that the sequences $\{ x_{n} \}$, $\{ y_{n} \}$ and $\{ u_{n} \}$ converge strongly to 0.

In Example 5.2, we give computer programming to support our main result.

### Example 5.2

Let $H_{1} = H_{2} = C = Q = \mathbb{R}^{2}$ be the two-dimensional Euclidean space of the real number with an inner product $\langle \cdot, \cdot \rangle: \mathbb{R}^{2} \times \mathbb{R}^{2} \rightarrow \mathbb{R}$ be defined by $\langle x, y \rangle = x \cdot y = x_{1}y_{1}+x_{2}y_{2}$ where $x=(x_{1}, x_{2}) \in \mathbb{R}^{2}$ and $y=(y_{1}, y_{2}) \in \mathbb{R}^{2}$ and a usual norm $\Vert \cdot \Vert : \mathbb{R}^{2} \rightarrow \mathbb{R}$ be defined by $\Vert x \Vert = \sqrt{x_{1}^{2}+x _{2}^{2}}$ where $x=(x_{1}, x_{2}) \in \mathbb{R}^{2}$. Let the mapping $A : \mathbb{R}^{2} \rightarrow \mathbb{R}^{2}$ be defined by $Ax = (2x_{1}-x_{2}, x_{1}+2x_{2})$ for all $x=(x_{1}, x_{2}) \in \mathbb{R}^{2}$ and $A^{*} : \mathbb{R}^{2} \rightarrow \mathbb{R} ^{2}$ be defined by $A^{*}z = (2z_{1}-z_{2}, 2z_{2}-z_{1})$ for all $z=(z_{1}, z_{2}) \in \mathbb{R}^{2}$. Let $D_{1},D_{2} : \mathbb{R} ^{2} \rightarrow \mathbb{R}^{2}$ be defined by $D_{1}x = ( \frac{x _{1}}{6}, \frac{x_{2}}{6} ) $ and $D_{2}x = ( \frac{x_{1}}{2}, \frac{x_{2}}{3} )$, $\forall x = (x_{1}, x_{2}) \in \mathbb{R}^{2}$, respectively. Let the mapping $T_{i}: \mathbb{R} ^{2} \rightarrow \mathbb{R}^{2}$ be defined by $T_{i}x = ( \frac{3ix _{1}}{3i+1}, \frac{3ix_{2}}{3i+2}{} )$, $\forall x = (x_{1}, x _{2}) \in \mathbb{R}^{2}$ and $k_{i} = \frac{6}{7^{i}} + \frac{1}{N7^{N}}$ for every $i = 1, 2, \ldots, N$. Let the mapping $F_{1}, F_{2} : \mathbb{R}^{2}\times \mathbb{R}^{2} \rightarrow \mathbb{R}$ be defined by

$$F_{1}(x, y) = -x^{2}+y^{2},\quad \forall x = (x_{1}, x_{2}), y = (y_{1}, y _{2})\in \mathbb{R}^{2}, $$

and

$$F_{2}(x, y) = -2x^{2}+xy+y^{2},\quad \forall x = (x_{1}, x_{2}), y = (y _{1}, y_{2})\in \mathbb{R}^{2}. $$

Let the mapping $f_{1}: \mathbb{R}^{2} \rightarrow \mathbb{R}^{2}$ be defined by $f_{1}x = ( \frac{x_{1}}{5}, \frac{x_{2}}{5} )$, $\forall x = (x_{1}, x_{2})\in \mathbb{R}^{2}$ and the mapping $f_{2}: \mathbb{R}^{2} \rightarrow \mathbb{R}^{2}$ be defined by $f_{2}x = ( \frac{x_{1}}{7}, \frac{x_{2}}{7} )$, $\forall x = (x_{1}, x_{2})\in \mathbb{R}^{2}$. Let $r=1$ and $s=0.5$, the sequences $z_{n}= (z_{n}^{1}, z_{n}^{2})$, $x_{n}=(x_{n}^{1}, x_{n}^{2})$, $u_{n}=(u _{n}^{1}, u_{n}^{2})$, $y=(y_{1}, y_{2})\in \mathbb{R}^{2}$. By the definition of $f_{1}$ and $f_{2}$, we get

$$\begin{aligned} 0 \leq {}& F_{1} ( z_{n},y ) + \bigl\langle f_{1} ( z_{n} ), y-z_{n} \bigr\rangle + \frac{1}{r} \langle y-z_{n}, z_{n}-x_{n} \rangle \\ = {}& - \bigl( z_{n}^{1} \bigr) ^{2}- \bigl( z_{n}^{2} \bigr) ^{2}+ ( y _{1} ) ^{2}+ ( y_{2} ) ^{2}+\frac{1}{5}z_{n}^{1} \bigl( -z _{n}^{1}+y_{1} \bigr) + \frac{1}{5}z_{n}^{2} \bigl( -z_{n}^{2}+y_{2} \bigr) \\ &{}+ \bigl( y_{1}-z_{n}^{1} \bigr) \bigl( z_{n}^{1}-x_{n}^{1} \bigr) + \bigl( y_{2}-z_{n}^{2} \bigr) \bigl( z_{n}^{2}-x_{n}^{2} \bigr) \\ = {}& \biggl( ( y_{1} ) ^{2}+ \biggl( -x_{n}^{1}+ \frac{6}{5}z_{n} ^{1} \biggr) y_{1}+x_{n}^{1}z_{n}^{1}- \frac{11}{5} \bigl( z_{n}^{1} \bigr) ^{2} \biggr) \\ &{}+ \biggl( ( y_{2} ) ^{2}+ \biggl( -x_{n}^{2}+ \frac{6}{5}z_{n} ^{2} \biggr) y_{2}+x_{n}^{2}z_{n}^{2}- \frac{11}{5} \bigl( z_{n}^{2} \bigr) ^{2} \biggr) \\ = {}& G_{1} ( y_{1} ) +G_{2} ( y_{2} ). \end{aligned}$$

Let $G_{1}(y_{1})= ( y_{1} ) ^{2}+ ( -x_{n}^{1}+ \frac{6}{5}z_{n}^{1} ) y_{1}+x_{n}^{1}z_{n}^{1}-\frac{11}{5} ( z_{n}^{1} ) ^{2}$ and $G_{2}(y_{2})= ( y_{2} ) ^{2}+ ( -x _{n}^{2}+\frac{6}{5}z_{n}^{2} ) y_{2}+x_{n}^{2}z_{n}^{2}- \frac{11}{5} ( z_{n}^{2} ) ^{2}$. $G_{1}(y_{1})$ and $G_{2}(y_{2})$ are quadratic functions with coefficients $a_{1} =1$, $b_{1} = -x_{n}^{1}+\frac{6}{5}z_{n}^{1}$, and $c_{1} = x_{n}^{1}z _{n}^{1}-\frac{11}{5} ( z_{n}^{1} ) ^{2}$ of $G_{1}(y_{1})$ and coefficients $a_{2} =1$, $b_{2} = -x_{n}^{2}+\frac{6}{5}z_{n}^{2}$, and $c_{2} = x_{n}^{2}z_{n}^{2}-\frac{11}{5} ( z_{n}^{2} ) ^{2}$ of $G_{2}(y_{2})$, respectively. Determine the discriminant $\Delta_{1}$ of $G_{1}$ as follows:

$$\begin{aligned} \Delta_{1} = {}& b_{1}^{2} - 4a_{1}c_{1} \\ = {}& \biggl( -x_{n}^{1}+\frac{6}{5}z_{n}^{1} \biggr) ^{2}-4(1) \biggl( x _{n}^{1}z_{n}^{1}- \frac{11}{5} \bigl( z_{n}^{1} \bigr) ^{2} \biggr) = \frac{1}{25} \bigl( 5x_{n}^{1}-16z_{n}^{1} \bigr) ^{2}. \end{aligned}$$

We know that $G_{1}(y_{1}) \geq 0$, $\forall y \in \mathbb{R}$. If it has most one solution in $\mathbb{R}$, then $\Delta_{1} \leq 0$, so we obtain $z_{n}^{1} = \frac{5x_{n}^{1}}{16}$. Next, we determine the discriminant $\Delta_{2}$ of $G_{2}$ by using the same method as above, we obtain $z_{n}^{2} = \frac{5x_{n}^{2}}{16}$. That is $T^{F_{1}}_{r} ( I-rf _{1} ) z_{n}= ( \frac{5x_{n}^{1}}{16}, \frac{5x_{n}^{2}}{16} ) $. After that, we find the solution of $u_{n}= ( u_{n}^{1},u_{n}^{2} ) $ in this inequality $0 \leq F_{2} ( u_{n},y ) + \langle f_{2} ( u_{n} ), y-u_{n} \rangle + \frac{1}{s} \langle y-u_{n}, u_{n}-x_{n} \rangle $. By using the same method as $z_{n}= (z_{n}^{1}, z_{n} ^{2})$, we obtain

5.1 $$ u_{n} = \bigl( u_{n}^{1}, u_{n}^{2} \bigr) = \biggl( \frac{7x_{n}^{1}}{51}, \frac{7x _{n}^{2}}{51} \biggr). $$

That is, $T^{F_{2}}_{s} ( I-sf_{2} ) u_{n}= ( \frac{7x _{n}^{1}}{51}, \frac{7x_{n}^{2}}{51} ) $.

Let $x_{1}=(x_{1}^{1}, x_{1}^{2})$ and $u=(u_{1}, u_{2})\in \mathbb{R}^{2}$. The sequences $\{ x_{n} \} $, $\{ y_{n} \} $ and $\{ u_{n} \} $ are generated by (3.6), where $k_{i}=\frac{6}{7^{i}}+ \frac{1}{N7^{N}}$, $d_{1}=1$, $d_{2}=1$, $a=0.5$, $\alpha_{n}=\frac{1}{2n}$, $\beta_{n}=\frac{7n-4}{12n}$, $\gamma_{n}=\frac{5n-2}{12n}$ and $\lambda_{n}=\frac{1}{2n^{2}}$ for all $n \in \mathbb{N}$. Since $L=5$, we choose $\gamma =0.1$. From the definition of $D_{1}$, $D_{2}$, $T_{i}$, $F_{1}$, $F_{2}$, $f_{1}$ and $f_{2}$, we have $VI(C,D_{1}) \cap VI(C,D _{2}) \cap \bigcap^{N}_{i=1} F ( T_{i} ) \cap \Omega =\{0\}$. From Theorem 3.4, we can conclude that the sequences $\{ x_{n} \}$, $\{ y_{n} \}$ and $\{ u_{n} \}$ converge strongly to 0. We can rewrite (3.6) as follows:

$$\textstyle\begin{cases} \textstyle\begin{array}{l l l} u_{n} = T^{F_{1}}_{1} ( I-f_{1} ) ( x_{n}+0.1A^{*} ( T^{F_{2}}_{0.5} ( I-0.5f_{2} ) -I ) Ax_{n} ), \\ y_{n} = ( I-D_{1} ) ( 0.5u_{n}+0.5 ( I-D_{2} ) u _{n} ), \\ x_{n+1} = \frac{1}{2n}u + \frac{7n-4}{12n}x_{n} + \frac{5n-2}{12n} ( y_{n} - ( (\frac{1}{2n^{2}}) ( \sum^{N}_{i=1}( \frac{6}{7^{i}}+\frac{1}{N7^{N}}) ( y_{n}-T_{i}y_{n} ) ) ) ), \end{array}\displaystyle \end{cases} $$

for all $n\geq 1$, where $x_{n}=(x_{n}^{1}, x_{n}^{2})$, $y_{n}=(y_{n} ^{1}, y_{n}^{2})$ and $u_{n}=(u_{n}^{1}, u_{n}^{2})$.

Table 1 shows the values of sequences $\{ x _{n} \} $, $\{ y_{n} \} $ and $\{ u_{n} \} $ where $u=(5,-5)$, $x_{1}=(5,-5)$ and $n = 30$.

**Table 1:** The values of $\{x_{n}\}$, $\{y_{n}\}$ and $\{u_{n}\}$ where $u=(5,-5)$, $x_{1}=(5,-5)$ and $n = 30$

	*N* = 1			*N* = 20		
*n*	$x_{n}=(x_{n}^{1}, x_{n}^{2})$	$y_{n}=(y_{n}^{1}, y_{n}^{2})$	$u_{n}=(u_{n}^{1}, u_{n}^{2})$	$x_{n}=(x_{n}^{1}, x_{n}^{2})$	$y_{n}=(y_{n}^{1}, y_{n}^{2})$	$u_{n}=(u_{n}^{1}, u_{n}^{2})$
1	(5.0000, −5.0000)	(0.5553, −0.6170)	(0.8885, −0.8885)	(5.0000, −5.0000)	(0.5553, −0.6170)	(0.8885, −0.8885)
2	(3.8715, −3.8850)	(0.4300, −0.4794)	(0.6879, −0.6903)	(3.8700, −3.8833)	(0.4298, −0.4792)	(0.6877, −0.6901)
⋮	⋮	⋮	⋮	⋮	⋮	⋮
15	(0.5189, −0.5274)	(0.0576, −0.0651)	(0.0922, −0.0937)	(0.5189, −0.5274)	(0.0576, −0.0651)	(0.0922, −0.0937)
⋮	⋮	⋮	⋮	⋮	⋮	⋮
29	(0.2485, −0.2522)	(0.0276, −0.0311)	(0.0442, −0.0448)	(0.2485, −0.2522)	(0.0276, −0.0311)	(0.0442, −0.0448)
30	(0.2397, −0.2432)	(0.0266, −0.0300)	(0.0426, −0.0432)	(0.2397, −0.2432)	(0.0266, −0.0300)	(0.0426, −0.0432)

## Conclusion

1. Example 5.1 is an example in infinite dimensional Hilbert space for supporting Theorem 3.4
2. Table 1 and Fig. 1 in Example 5.2 show that the sequences $\{x_{n}\}$, $\{y_{n}\}$ and $\{u_{n}\}$ converge to 0, where $\{ 0 \} = VI(C,D_{1}) \cap VI(C,D_{2}) \cap \bigcap^{N}_{i=1} F ( T_{i} ) \cap \Omega $.

**Figure 1:**
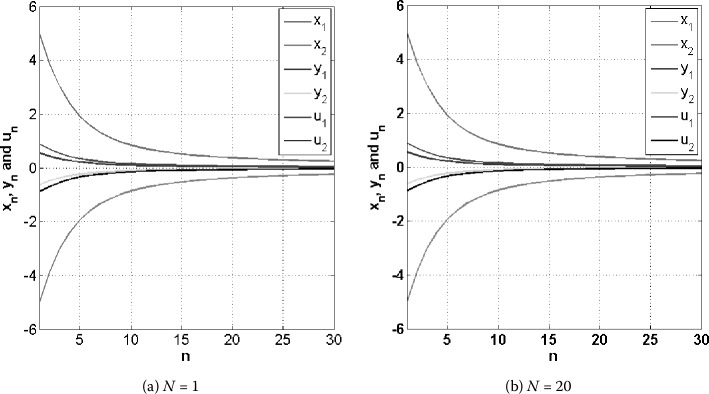
The convergence comparison with different values *N*
3. Theorem 3.4 guarantees the convergence of $\{x_{n}\}$, $\{y_{n}\}$ and $\{u_{n}\}$ in Example 5.1 and Example 5.2.
4. By using the concept of Picard iteration, Wang [13] defined the iterative scheme $\{x_{n}\}$ for solving SCFPP as follows:
  6.1 $$\begin{aligned} x_{n+1} &=x_{n}-\rho_{n} \bigl( ( I-U ) x_{n}+A^{*}(I-T)Ax_{n} \bigr) \\ &= \bigl( I-\rho_{n} \bigl( ( I-U ) +A^{*}(I-T)A \bigr) \bigr) x _{n}, \end{aligned}$$
  where $\rho _{n}$ is according to (1.4) and *U* and *T* are firmly quasi-nonexpansive mappings. Then the sequence $\{x_{n}\}$ converges weakly to *z*, where $z=\lim_{n \rightarrow \infty }P_{\Phi }x_{n}$. In Theorem 3.4, we use the concept of Halpern iteration and suitable conditions of the parameters $d_{1}$, $d_{2}$, *r*, *s*, *a*, *γ*, *L*, $\{ \alpha _{n} \}$, $\{ \beta _{n} \}$ and $\{ \gamma _{n} \}$, the sequence $\{x_{n}\}$ defined by (3.6) converges strongly to $z=P_{\mathcal{F}}u$, which is a different method from (6.1).
